# Exosomes of pasteurized milk: potential pathogens of Western diseases

**DOI:** 10.1186/s12967-018-1760-8

**Published:** 2019-01-03

**Authors:** Bodo C. Melnik, Gerd Schmitz

**Affiliations:** 1 Department of Dermatology, Environmental Medicine and Health Theory, University of Osnabrück, Am Finkenhügel 7A, 49076 Osnabrück, Germany; 2 Institute for Clinical Chemistry and Laboratory Medicine, University Hospital Regensburg, University of Regensburg, Josef-Strauss-Allee 11, 93053 Regensburg, Germany

**Keywords:** B cell lymphoma, Breast cancer, Cow’s milk, Diabetes mellitus, Exosomes, Hepatocellular carcinoma, MicroRNA, Obesity, Osteoporosis, Parkinson’s disease, Prostate cancer

## Abstract

Milk consumption is a hallmark of western diet. According to common believes, milk consumption has beneficial effects for human health. Pasteurization of cow’s milk protects thermolabile vitamins and other organic compounds including bioactive and bioavailable exosomes and extracellular vesicles in the range of 40–120 nm, which are pivotal mediators of cell communication via systemic transfer of specific micro-ribonucleic acids, mRNAs and regulatory proteins such as transforming growth factor-β. There is compelling evidence that human and bovine milk exosomes play a crucial role for adequate metabolic and immunological programming of the newborn infant at the beginning of extrauterine life. Milk exosomes assist in executing an anabolic, growth-promoting and immunological program confined to the postnatal period in all mammals. However, epidemiological and translational evidence presented in this review indicates that continuous exposure of humans to exosomes of pasteurized milk may confer a substantial risk for the development of chronic diseases of civilization including obesity, type 2 diabetes mellitus, osteoporosis, common cancers (prostate, breast, liver, B-cells) as well as Parkinson’s disease. Exosomes of pasteurized milk may represent new pathogens that should not reach the human food chain.

## Introduction

**Fig. 1:**
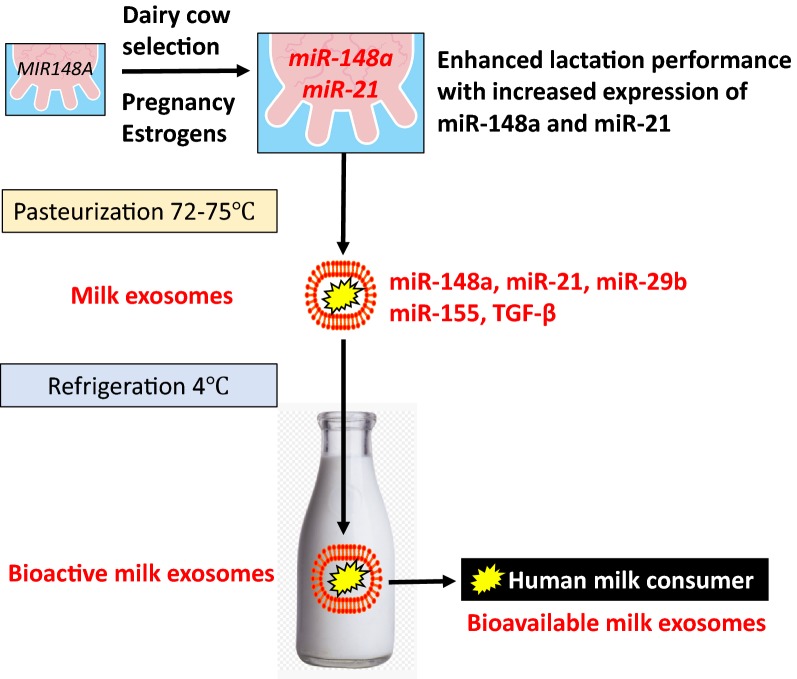
Transfer of dairy milk exosomes to the human milk consumer. Genetic dairy cow selection enhances mammary epithelial cell miR-148a expression, a crucial epigenetic mechanism enhancing milk yield that potentially also increases milk exosome miR-148a content. Persistent pregnancy of dairy cows further promotes estrogen-stimulated expression of miR-148a and miR-21. Milk exosomes also contain miR-155 and transforming growth factor-β (TGF-β), which promotes the expression of miR-155. Pasteurization has no significant effect on milk exosome integrity and exosomal miR bioavailability. Large scale pasteurization and cooling technology promoted the persistent entry of dairy milk exosomes and their miRs into the human food chain

Exosomes (40–120 nm) are members of a larger spectrum of extracellular vesicles (EVs) of up to 1000 nm that mediate cell-to-cell communication and cell function [1–4]. Milk exosomes and milk microvesicles (MVs) are released from mammary gland epithelial cells (MECs) of all mammals including humans and dairy cows [5–9]. Exosomes are formed from inward budding of endosomes resulting in membrane-surrounded multivesicular bodies (MVBs), which are secreted by fusion of the MVBs with the cell membrane. MVs are released directly by budding of the plasma membrane like milk fat globules (MFGs). Both pathways are highly regulated and appear to be conserved amongst different species [10]. In 2013, Melnik et al. [11] postulated that “milk is not just food” but a genetic transfection system activating mechanistic target of rapamycin complex 1 (mTORC1) signaling and microRNA (miR) transfer for postnatal growth. Today, compelling evidence confirms this *functional hypothesis of milk signaling.* Milk’s exosomal miRs serve as a biomolecular software for maternal-neonatal communication which is important for epigenetic gene regulation that is required for developmental processes of the newborn infant [12]. Abundantly present miRs in milk-derived EVs including miR-148a are highly conserved between mammals [13]. Various exosome-specific proteins, lipids, mRNAs, circular RNAs, non-coding miRs and regulatory proteins such as transforming growth factor-β (TGF-β) are crucial signaling components delivered by milk exosomes [5, 6, 14, 15]. Evidence has been provided that breast milk exosomes and their miR cargo play a key role for the appropriate maturation of the intestine, development of the gut microbiome and programming of the intestinal mucosa-associated lymphatic tissue (MALT) as well as thymic T cell differentiation [16–26]. The deficiency of milk exosomes in artificial formulas increases the risk for inappropriate metabolic and immunological programming of the newborn infant [8, 9, 18, 19], a major determinant for the development of diseases of civilization in later life such as allergic diseases and obesity [18, 19]. Under physiological conditions, the transfer of milk-derived exosomes and their miR-mediated impact on epigenetic regulation is restricted to the period of maternal lactation in all mammals, except Neolithic humans, who are exposed to dairy milk exosomes after the nursing period for several decades. Since the 1950s, when widely available refrigeration technology allowed the distribution of pasteurized milk and milk products, bioactive bovine milk exosomes entered the human food chain in a large scale (Fig. 1). It is the intention of this review article to provide epidemiological and translational evidence that dairy milk-derived exosomes and their cargo contribute to the pathogenesis of common diseases of civilization and should thus be regarded as critical pathogens, that have to be eliminated from the human food chain.

## Dairy milk exosomes and their miR cargo are bioavailable for the milk consumer

Reinhardt et al. [27] characterized the proteome of bovine milk exosomes and reported a greatly reduced presence of MFG membrane (MFGM) proteins in the fraction of cow milk exosomes, which suggests that milk exosome secretion pathways originate from Golgi and differ from that of MFGs, which resemble holocrine secretion of lipid droplets directly from the endoplasmic reticulum (ER). Bovine milk exosomes (50–100 nm) isolated by ultracentrifugation from the 100,000×*g* pellet from the milk of mid-lactation Holstein cows are enriched in tumor susceptibility gene-101 (TSG101), a protein component of the vesicular trafficking process and depleted in MFGM proteins such as lactaderin/MGFE8 [26]. Benmoussa et al. [28] confirmed that cow milk exosomes of the 100,000×*g* pellet fraction are positive for the exosome markers TSG101, apoptosis-linked gene 2-interacting protein X (ALIX), heat shock protein 70 (HSP70) and contain bovine miR-223 and miR-125b. A large quantity of bovine milk miR-223 and miR-125b resisted digestion under simulated gastrointestinal tract conditions, which supports their bioaccessibility [28]. Recently, a subset of milk MVs (100 nm in diameter) with proteins commonly found in MFGM has been characterized that sediments at low speed ultracentrifugation (35,000×*g*) and contains and protects the bulk of milk miRs from degradation [29, 30]. At present, there is a lack of information on the potential systemic biological effects and trafficking characteristics of this 35 K subset of milk EVs to the milk consumer.

This review focuses on milk exosomes of the 100,000×*g* fraction (100 K). It is generally appreciated that exosomes participate in cell-to-cell communication and gene regulation, facilitated by the transfer of miRs, proteins and lipids from donor to recipient cells. Bovine milk exosomes contain nearly 400 miRs and selected proteins [31–34] that resist the harsh conditions in the gastrointestinal tract [14, 28, 32], are taken up via receptor-mediated endocytosis by intestinal epithelial cells [35], vascular endothelial cells [36], and reach distant tissues across species boundaries after oral administration [35–39]. The most sophisticated and extensively controlled study of Manca et al. [39] recently demonstrated that bovine milk exosomes derived from commercial pasteurized skim milk reached the systemic circulation of mice and distributed widely among murine tissues. A variety of different tracers used in their study suggests that milk exosomes and their miR cargo accumulate in the brain, an important finding, which is consistent with proven exosomal delivery of Cre-recombinase to the brain [40]. Bovine miRs were analyzed by RNase H2-dependent PCR (rhPCR) in plasma collected from 11 healthy volunteers before and 6 h after consumption of 1.0 L of commercial 1%-fat cow’s milk. This method (rhPCR) is able to distinguish between bovine and human miRs with small variations in the nucleotide sequence. Notably, plasma concentrations of *Bos taurus* (bta)-miR-21-5p and bta-miR-30a-5p were > 100% higher 6 h after milk consumption than before milk intake, a finding confirming the bioavailability of dairy milk exosomes in humans [41]. The majority of dairy milk miRs including miR-148a, miR-21, miR-29b and miR-155 survive pasteurization and refrigerated storage but are significantly reduced after boiling or ultra-heat treatment (UHT) [32, 42–45]. Baier et al. [46] demonstrated the bioavailability of milk-borne miRs in humans using commercial milk (1% fat) that contained 148 ± 42 pmol/L of miR-29b. In a dose-dependent manner, human volunteers absorbed considerable amounts of miR-29b from cow milk resulting in a plasma peak of miR-29b at about 4 h to 6 h postprandial associated with an intracellular increase of miR-29b in peripheral blood mononuclear cells (PBMC) [46]. Furthermore, it has been demonstrated that bovine milk exosomes are taken up by human macrophages [47].

Recent evidence underlines that bacterial fermentation of milk decreases the size, protein- and miR content of milk exosomes [48]. It has been demonstrated that milk-derived exosomes are taken up by *Escherichia coli* K-12 MG1655 and *Lactobacillus plantarum* WCFS promoting bacterial growth [49]. In contrast to pasteurization (78 °C), boiling (100 °C), and ultra-heat treatment (130 °C) of milk decreased the levels of milk miRs [43, 44].

Taken together, there is compelling evidence that dairy milk exosomes of pasteurized commercial milk reach the systemic circulation and tissues of the human milk consumer. Whereas human breast milk-derived exosomes are of critical importance for infant health and appropriate development, programming and tissue maturation [5–7, 11, 16–26], continued exposure of humans to dairy milk-derived exosomes after the nursing period may exert adverse effects on human health.

## Allergic diseases

Exosomes and exosomal miR signaling play a key role during postnatal programming and tissue maturation of the infant [5–9, 50]. Breastfeeding has a protective effect on the prevention of allergic rhinitis, allergic asthma and atopic dermatitis [51]. Breastfeeding in contrast to commercial artificial formula feeding is regarded as the most efficient primary prevention of allergic asthma in childhood [52–55]. Allergy-prone and allergic individuals exhibit reduced numbers and function of regulatory T cells (Tregs) [56]. Forkhead box P3 (FoxP3) is the master transcription factor of Tregs and controls Treg differentiation and maintenance of Treg-mediated immune tolerance [57–59]. Tooley et al. [60] demonstrated that maternal rat milk, but not formula, prevented β-lactoglobulin-induced allergy in rat pups. Thus, maternal milk in contrast to formula contains an ingredient conferring an allergy-preventive effect. Notably, Admyre et al. [16] showed that the addition of human breast milk exosomes to PBMCs increased the number of FoxP3^+^CD4^+^CD25^+^ Tregs in a dose-dependent manner. *FOXP3* gene expression is controlled by epigenetic mechanisms as well as TGF-β [61–63]. The Treg-specific demethylation region (TSDR) is a critical region of the *FOXP3* promoter, which controls FoxP3 expression. TSDR methylation reduces FoxP3 expression, whereas TSDR demethylation promotes FoxP3 expression, respectively [61, 62]. In allergic individuals, an increased TSDR methylation has been observed [64, 65]. In contrast, tolerance induction and cessation of allergy was associated with TSDR *FOXP3* demethylation [65, 66]. Both DNA methyltransferase 1 (DNMT1) and DNMT3B are associated with the *FOXP3* locus in CD4^+^T cells [67]. miR-148a directly targets DNMT1, whereas miR-21 indirectly downregulates DNMT1 expression by targeting an important autoimmune gene, *Ras guanyl nucleotide*-*releasing protein 1* (RASGRP1), which mediates the Ras-MAPK pathway upstream of DNMT1 [68]. miR-29b is another miR species that negatively regulates DNMT1 expression [69–71]. Importantly, miR-148a, miR-21, miR-29b and miR-155 are cargos of human and bovine milk exosomes [17, 23, 35, 45, 48, 72]. Golan-Gerstl et al. [44] demonstrated that incubation of breast milk exosomes with intestinal cells increased their miR-148a content resulting in decreased expression of DNMT1. Importantly, miR levels in dairy milk were not significantly affected by pasteurization [43–45].

**Fig. 2:**
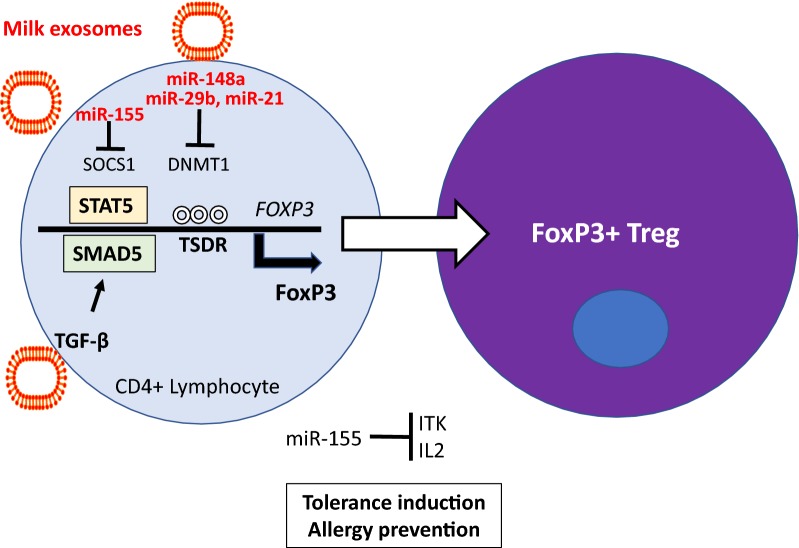
Milk exosomes and allergy prevention. Human breast milk and dairy milk exosomes transfer miR-148a and miR-29b, which both suppress DNA methyltransferase 1 (DNMT1). DNMT1 controls the methylation status of the Treg-specific demethylation region (TSDR) on the *FOXP3* promoter. DNMT1 suppression (TSDR hypomethylation) increases FoxP3 expression. Milk exosome-derived miR-155 inhibits suppressor of cytokine signaling 1 (SOCS1), a negative regulator of the JAK-STAT pathway that increases the expression of signal transducer and activator of transcription 5 (STAT5) promoting FoxP3 expression. Milk exosome-derived transforming growth factor-β (TGF-β) enhances SMAD5 signaling that further increases FoxP3 expression, especially in the thymus. Milk exosomes thus promote the induction of FoxP3, the master transcription factor of regulatory T cells (Tregs), the potential mechanism preventing allergy development by breast feeding or raw farm milk consumption during early infancy

Consumption of raw cow milk during early infancy exhibited a preventive effect on the development of allergic diseases and increased the number of FoxP3 + Tregs [73–75]. The existence of a postnatal window for milk-induced Treg maturation has been proposed [76]. We hypothesized that thymic maturation of Tregs is mediated by milk-derived exosomes [18, 19]. miR-155 plays a critical role in the maturation of thymic Tregs [18, 19]. miR-155 targets *suppressor of cytokine signaling 1* (SOCS1), a critical inhibitor of *signal transducer and activator of transcription 5* (STAT5), which promotes the expression of FoxP3 [77]. Higher amounts of SOCS1 protein suppress IL-12 and IFNγ signaling inhibiting Th1 cell differentiation, while promoting Th2 cell induction [78, 79]. TGF-β as well promotes thymic Treg (tTreg) cell development by inducing FoxP3 expression repressing T cell clonal deletion and peripheral Treg cell differentiation [63, 80]. Notably, bovine milk exosomes contain both miR-155 and TGF-β [14, 32, 45], a fact, that further supports milk exosome-driven maturation of tTregs [18, 19]. It is likely that milk exosomes or exosome-derived molecules, which reach distant tissue including the brain may also accumulate in the thymus [39], an organ exhibiting extensive exosome traffic [81–83]. Milk exosomes may thus support thymic epithelial cell-derived exosomes in tTreg differentiation and maturation, a potential augmenting mechanism which may explain allergy prevention early in life by consumption of raw cow’s milk, during a period when the infant’s thymus is still functional operative (Fig. 2).

In addition, milk exosomes may have a direct effect on MALT homeostasis. TGF-β, a component of milk exosomes [14], has been shown to induce miR-155 in both freshly isolated and lamina propria T cell lymphoblasts [84]. miR-155 targets IL-2 inducible T-cell kinase (ITK) and decreases ITK and IL-2 mRNA suggesting a TGF-β-dependent function for miR-155 in modulating cytokine and T-cell immune responses in the gut [84]. Intriguingly, the concentration of TGF-β1 in colostrum samples from mothers of infants with IgE-mediated cow’s milk allergy (CMA) was significantly lower than from mothers of infants with non-IgE-mediated CMA [85] pointing to an important role of TGF-β/miR-155 signaling in intestinal immune homeostasis.

In contrast to breastfeeding, artificial infant formula powder contains no bioactive exosomes and only minor amounts of exosomal TGF-β and miRs (for instance < 10% of miR-148a) compared to raw cow’s milk [32, 86]. This may be a reasonable explanation for the superiority of breastfeeding in allergy prevention compared to formula feeding [18, 19]. These data strongly indicate that milk exosomes are of critical importance for the maturation of the immune system during the postnatal period and early infancy.

## Fetal macrosomia

The *Developmental Origins of Health and Disease (DOHaD)* hypothesis underlines the impact of prenatal and postnatal epigenetic factors in the transmission of obesity and cardiovascular diseases [87, 88]. Accelerated fetal growth and increased birth weight are well-known risk factors for the development of obesity and T2DM [89–91]. Disturbances of the intrauterine milieu can induce lifelong deviations of metabolic programming [92]. Exosomes have been identified as key players for fetal-maternal communication and vice versa [93]. As milk exosomes and their cargo products are able to overcome tissue barriers including the intestinal and blood–brain barrier and are distributed in various tissues [39], it is conceivable that they may also reach the placenta of women consuming pasteurized milk. Worldwide gynecological societies such as the *American College of Obstetricians and Gynecologists* recommend increased milk and dairy consumption during pregnancy as a rich source of calcium [94]. During 1996–2002, the *Danish National Birth Cohort* collected data on midpregnancy diet of 50,117 mother-infant-pairs and ascertained birth outcomes [95]. This study demonstrated that increased milk consumption during pregnancy was associated with an increase in placental and birth weight [95]. Maternal milk consumption, fetal growth, and the risks of neonatal complications have been investigated in the *Generation R Study* in Rotterdam including 3405 mothers [96]. Maternal milk consumption of > 3 glasses/day was associated with greater fetal weight gain in the third trimester of pregnancy, which led to an 88 g higher birth weight than that with milk intake of none to 1 glass/day. In addition, head circumference tended to be 2.3 cm larger when mothers consumed > 3 glasses/day. This association appeared to be limited only to milk, whereas protein intake from non-dairy food or cheese was not associated with an increase in birth weight [96]. A systematic review of all studies and case reports supported the conclusion that only milk consumption but not the intake of fermented milk/milk products increased birth weight [97, 98]. Thus, some compounds of unfermented milk not related to milk proteins, accelerate fetal growth.

**Fig. 3:**
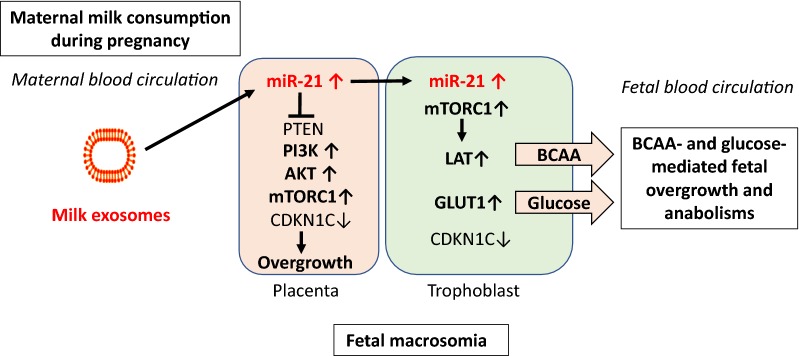
Dairy milk exosomes and fetal macrosomia. Milk exosome-derived miR-21 may increase placental miR-21 content promoting mTORC1 signaling via inhibition of phosphatase and tensin homolog (PTEN) and other regulatory checkpoints. Increased mTORC1-mediated placental growth enhances the nutrient transfer to the fetus. In the trophoblast, upregulated mTORC1 increases the expression of L-type amino acid transporters (LAT) and glucose transporter 1 (GLUT1), thus overstimulating the diaplacental flux of branched-chain amino acids (BCAAs) and glucose to the fetus promoting fetal overgrow (macrosomia). miR-21 also targets CDKN1C, a critical checkpoint for fetal growth mutated in Beckwith-Wiedemann syndrome

Two independent studies confirmed increased expression of miR-21 in placenta tissue of infants born with macrosomia (birth weight > 4000 g) [99, 100]. Milk miR-21, a signature miR of commercial dairy milk and cargo of bovine milk exosomes [71, 72, 80], most likely reaches the placenta and increases placental and trophoblast growth. miR-21 plays important roles in growth of trophoblastic cell lines [101] and increases PI3K-AKT- and mTORC1 signaling by suppression of various key inhibitory checkpoints such as PTEN [11]. Stimulation of trophoblast mTORC1 activity enhances the transfer of branched-chain amino acids (BCAAs) to the fetus, a constellation that may promote BCAA-mTORC1-driven fetal macrosomia [102–107] (Fig. 3). About 5–10% of cases exhibiting Beckwith-Wiedemann syndrome (BWS), an overgrowth syndrome characterized by macrosomia, macroglossia, and abdominal wall defects, are caused by loss-of-function mutations of *cyclin*-*dependent kinase inhibitor 1C* (CDKN1C, p57kip2) [108–112]. CDKN1C is the cyclin-dependent kinase inhibitor of G1 cyclin complexes that functions as a negative regulator of cellular growth and proliferation [113]. Notably, miR-21 is one of several miRs that directly target CDKN1C [113], a further epigenetic mechanism linking dairy milk exosome intake during pregnancy to fetal macrosomia.

## Adipogenesis and obesity

The worldwide epidemic of obesity is a growing health problem, associated with increased risk of chronic diseases especially type 2 diabetes mellitus (T2DM). Young mice who had long-term ad libitum access to commercial whole cow’s milk in comparison to mice that received low fat milk or controls that had no access to dairy milk exhibited increased body weight and epididymal fat mass [114]. There is increasing interest in the role of exosomes and exosome-transferred miRs in the regulation of mesenchymal stem cell (MSC)-derived adipogenesis [115–120]. miRs regulate adipogenic lineage commitment in MSCs and hence govern fat cell numbers [115]. MSCs arise from a variety of tissues, including bone marrow and adipose tissue and, accordingly, have the potential to differentiate into multiple cell types, including osteoblasts and adipocytes [117]. An inverse relationship exists in adipogenic and osteogenic lineage commitment and differentiation, such that signaling pathways induce adipogenesis at the expense of osteogenesis and vice versa [117]. Peroxisome proliferator-activated receptor γ (PPARγ) is known to function as a master transcriptional regulator of adipocyte differentiation, but inhibits osteoblast differentiation [117]. In contrast, inducers of osteogenic differentiation, such as bone morphogenetic protein (BMP) and wingless-type MMTV integration site family members (Wnt), inhibit the function of PPARγ transactivation during MSC differentiation towards adipocytes [120]. Notably, MSCs differentiated on osteoblast extracellular matrix (ECM) with adipogenic exosomes showed expression of adipogenic lineage genes, while MSCs differentiated on adipocyte ECM with osteoblast exosomes showed osteogenic lineage genes [118]. These findings indicate that exosomes might override ECM-mediated instructive signals during lineage specification of MSCs [118]. Accumulating evidence indicates that miRs act as switches for MSCs to differentiate into either osteogenic or adipogenic lineages [120]. Based on these observations, it is conceivable that dairy milk-delivered exosomes and their miR cargo as well may interfere with MSC-derived adipogenesis and osteogenesis.

**Fig. 4:**
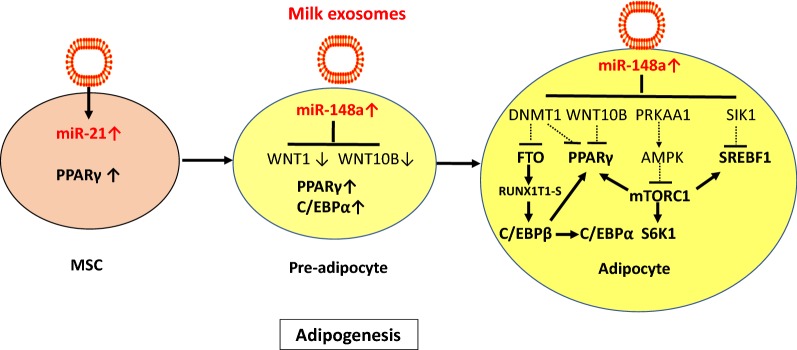
Dairy milk exosomes and adipogenesis. miR-21 induces the differentiation of mesenchymal stem cells (MSCs) towards adipocytes via activation of peroxisome proliferator-activated receptor PPARγ (PPARγ). miR-148a directly targets and suppresses the inhibitors of adipogenesis Wingless 1 (WNT1) and WNT10B increasing the expression of PPARγ and *CCAAT/enhancer binding protein α* (C/EBPα). miR-148a-mediated suppression of DNMT1 via promoter hypomethylation increases the expression of *fat mass and obesity*-*associated gene* (FTO), PPARγ and sterol regulatory element binding-transcription factor 1 (SREBF1). The mRNA demethylase FTO removes a m^6^A mark on RUNX1T1 mRNA generating its short splice variant RUNX1T1-S, which relieves RUNX1T1-mediated inhibition of C/EBPβ. Activated C/EBPβ activates the key adipogenic transcription factors C/EBPα and PPARγ. miR-148a targets PRKAA1, the catalytic α-unit of AMP-activated protein kinase (AMPK), the key negative regulator of mechanistic target of rapamycin complex 1 (mTORC1). mTORC1 activation enhances the expression of PPARγ and SREBF1, key lipogenic transcription factors. In addition, miR-148a targets *salt*-*inducible kinase 1* (SIK1), and thereby relieves its inhibitory action on SREBF1. Milk exosome-derived miR-148a is thus an adipogenesis promoting factor that operates at pivotal regulatory checkpoints enhancing the risk of obesity

miR-148a, a component of milk exosomes, is increased in adipose tissues from obese individuals and mice fed a high-fat diet (HFD) [121]. miR-148a suppresses its target gene Wnt1, an endogenous inhibitor of adipogenesis. Ectopic expression of miR-148a accelerated differentiation and partially rescued Wnt1-mediated inhibition of adipogenesis, whereas knockdown of miR-148a inhibited adipogenesis [121, 122]. In addition, miR-148a has been shown to silence Wnt10b, a further endogenous inhibitor of adipogenesis during 3T3-L1 cell differentiation [123]. A further study demonstrated that increased expression of miR-148a via suppression of DNMT1 enhanced adipocyte differentiation [124]. In the absence of DNMT1, adipocyte-specific gene expression and lipid accumulation occurred precociously [124]. Yang et al. [125] recently demonstrated that DNA methylation biphasically regulates 3T3-L1 preadipocyte differentiation [125]. Inhibition of DNA methylation at late stage of preadipocyte differentiation promoted lipogenesis and adipocyte phenotype in 3T3-L1 cells. This is likely mediated by induction of *sterol regulatory element*-*binding transcription factor 1c* (SREBF1c), whose promoter activity is upregulated by DNA demethylation during adipogenesis [125]. Persisting transfer of milk exosomal miR-148a may thus enhance SREBF1c-mediated lipid accumulation in adipocytes (Fig. 4). Remarkably, the *MIR148A* gene has been identified as an obesity risk gene in humans exhibiting single nucleotide polymorphisms which enhance miR-148a expression [126–128].

miR-21, another signature miR of bovine milk exosomes, is involved in adipocyte differentiation [129–132]. Kim et al. [129] showed that miR-21 governs human adipose tissue-derived MSC differentiation towards adipocytes. Furthermore, a correlation between miR-21 level and adipocyte numbers in the epididymal fat of mice fed a HFD has been observed [129]. Mei et al. [130] reported that overexpression of miR-21 in MSCs elevated the expression level of the differentiation-associated gene PPARγ, whereas miR-21 knockdown reduced PPARγ expression. miR-21 modulated ERK-MAPK activity by repressing Sprouty 2 (SPRY2), a known regulator of the receptor tyrosine kinase signaling pathway, that controls the magnitude of ERK-MAPK signaling during MSC differentiation [130]. Kang et al. [131] confirmed that miR-21 promotes adipocyte differentiation. It has recently been demonstrated that miR-21 expression was twofold greater in adipose tissue of patients with T2DM [132].

miR-29b, another abundant exosome-derived miR of cow’s milk, is also involved in adipogenesis [133]. During normal adipogenic differentiation of adipose tissue-derived stromal cells, upregulation of miR-29b promoted adipogenesis. Remarkably, miR-29 family members enhance lactation performance in dairy cow MECs via suppression of DNMT3A and DNMT3B. In contrast, inhibition of miR-29 s caused global DNA hypermethylation and increased the methylation levels of promoters of lactation-related genes, including casein αs1 (CSN1S1), E74-like factor 5 (ElF5), PPARγ, SREBF1, and glucose transporter 1 (GLUT1) and thereby reduced the secretion of lactoprotein, triacylglycerols and lactose by dairy cow MECs [133]. Thus, promoter demethylation of lipidogenic genes via miR-mediated DNMT suppression enhances both adipogenesis and lactation.

Overexpression of miR-155 in mice has been shown to reduce brown adipose tissue (BAT) mass [134]. Thus, milk exosome-derived miR-155 may attenuate BAT differentiation and thermogenesis via BAT, an unfavorable condition promoting lipid and energy storage in white adipose tissue (WAT) further promoting obesity.

## Hyperphagia

**Fig. 5:**
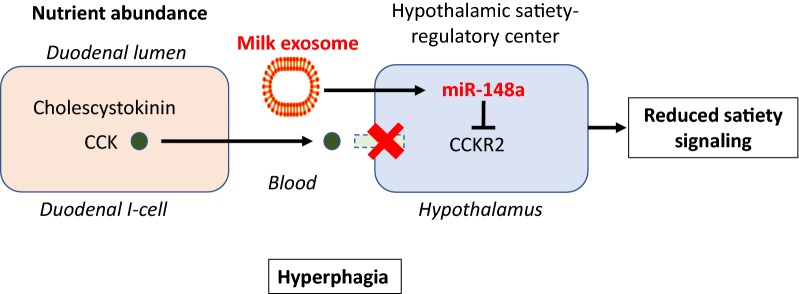
Milk exosomes and hyperphagia. Milk, a feeding and signaling system promoting postnatal anabolism and growth, most likely interferes with satiety control in the hypothalamus, which is possible as milk exosomes accumulate in the brain. Cholecystokinin (CCK) is released by duodenal I-cell during intestinal nutrient abundance. CKK is an important hormone that induces satiety signals in the hypothalamus via binding to CCK receptor 2 (CCKR2). CCKR2 is a direct target of miR-148a. It is thus conceivable that milk exosomes maintain a “hungry brain” to increase milk intake during the breastfeeding period. Persistent milk exosome intake by consumption of pasteurized cow’s milk may maintain this hyperphagic state, a further mechanism promoting obesity

Whole cow’s milk consumption in young mice not only increased body weight but also caloric intake [114]. The suppression of satiety signals during the period of lactation may be an intrinsic mechanism of milk signaling to enhance anabolism during the postnatal growth phase. As milk exosomes and their cargo products pass the blood–brain barrier and reach the brain [39, 135, 136], they may interfere with hypothalamic control centers of satiety feedback regulation. It has recently been shown that hypothalamic stem cells control ageing speed partly through exosomal miRs [137]. The brain-gut-axis is an interdependent system affecting neural functions and controlling eating behavior [138]. One of the hormones sending satiety signals to the hypothalamus is cholecystokinin (CCK), which is secreted from intestinal mucosa cells when the duodenum is filled with food [138]. CCK binds and signals via CCK1 receptor (CCK1R) and CCK2R. CCK2R knock out mice developed obesity that was associated with hyperphagia [139]. Suppression of feeding and concomitantly increased expression of hypothalamic proopiomelanocortin after intracerebroventricular injection of gastrin into control mice demonstrates that hypothalamic CCK2Rs mediate inhibition of food intake [139]. CCK2R deletion was associated with increased body weight and hypothalamic neuropeptide Y (NPY) content, which explains the increased food intake in CCK2R knockout mice [140]. Notably, the gene expressing CCK2R (*CCKBR*) is a direct target gene of miR-148a [141]. Thus, miR-148a of milk exosomes via suppression of satiety signals may maintain the state of a “hungry brain”, advantageous for postnatal growth but critical for long-term energy balance in adults (Fig. 5).

## Type 2 diabetes mellitus

T2DM is an increasing epidemic in developed countries and is closely linked to obesity (diabesity). Most epidemiological studies and systematic reviews promote the view that milk and dairy products are good for metabolic health and may reduce the risk of T2DM [142–147]. Dairy product intake may be inversely associated with risk of T2DM, but the evidence is inconclusive for total dairy products and sparse for individual types of dairy products. There are only few epidemiological studies that compare the risk of milk *versus* fermented milk/products. This, however, is of critical importance because fermentation of milk negatively affects the bioactivity of milk exosomes and their miR cargo [48]. The largest study that investigated the association of T2DM with intake of milk *versus* fermented milk products is the *European Prospective Investigation into Cancer and Nutrition* (n = 340,234) [148]. Whereas the consumption of fermented milk and fermented milk products confirmed an inverse relation to T2DM risk, the intake of non-fermented milk showed an increased risk for T2DM [148]. Furthermore, the *Physicians’ Health Study* (n = 21,660) demonstrated a significant increase in T2DM risk in relation to the consumption of whole milk [149]. Data presented from the *Framingham Heart Study Offspring Cohort* demonstrated a nonlinear correlation between milk consumption and prediabetes (defined by fasting glucose plasma levels ≥ 100 to < 126 mg/dl). More than 5 servings of milk per week significantly increased the risk of prediabetes [150]. None of these studies considered thermal milk processing (pasteurization versus UHT), which is of crucial importance for the bioavailability and function of exosomes in commercial milk products.

Milk protein provides abundant essential BCAAs including leucine, which activates mTORC1 [151], a key driver of anabolism, growth and insulin secretion [152]. Elevated BCAA plasma levels correlate with an increased risk of insulin resistance and T2DM [153–159].

Cow milk exosomes provide miR-29b, an abundant miR of dairy milk that survives pasteurization and exhibits the same nucleotide sequence as human miR-29b [42]. Consumption of pasteurized cow’s milk by healthy volunteers increased plasma levels of miR-29b including intracellular miR-29b levels in PBMCs [46]. Notably, diabetes researchers regard the miR-29s as a diabetogenic miR family [160–164]. Intriguingly, miR-29b controls the expression of *branched chain α*-*ketoacid dehydrogenase* (BCKD) complex in the cell via targeting the BCKD core protein *dihydrolipoamide branched*-*chain acyltransferase* (DBT) [165]. In accordance to a recent study, early-onset and classical forms of T2DM showed impaired expression of BCKD genes involved in muscle BCAA catabolism [166]. miR-29b-mediated inhibition of BCKD activity decreases BCAA catabolism, a meaningful metabolic switch for the newborn mammal. The essential BCAAs are required for the synthesis of many functional and structural proteins [167]. Therefore, BCAAs should not be wasted for purposes of energy generation during postnatal growth [154]. In addition, BCAAs play a key role as activators of mTORC1, which orchestrates cell growth and anabolism [168–171]. In cells with impaired leucine catabolism, mTORC1 signaling towards phosphorylation of ribosomal protein S6 kinase 1 (S6K1) was significantly increased [172].

Insulinotropic amino acids, especially leucine and glutamine, are amino acids highly enriched in milk proteins that are capable to increase insulin secretion [173–175]. Leucine supplementation in mice stimulated insulin secretion of pancreatic islets, which was associated with an activation of the PI3K/AKT/mTORC1 pathway [174]. Insulin has growth-promoting effects and binds to insulin- and insulin-like growth factor 1 (IGF-1) receptors. Insulin regulates appetite, body temperature, white fat mass, and glucose metabolism. Importantly, insulin signaling modulates neurotransmitter activity, neuronal function and synaptogenesis, critical events during the postnatal period [176]. Inhibition of BCKD by exosomal miR-29b may serve to increase β-cells BCAA levels further promoting mTORC1-mediated insulin secretion during the postnatal growth phase. However, this is a critical regulatory switch enhancing endoplasmic reticulum (ER) stress and β-cell apoptosis in the long run [151]. In fact, chronic exposure to leucine in vitro has been shown to induce β-cell dysfunction in INS-1E cells and mouse islets [177].

**Fig. 6:**
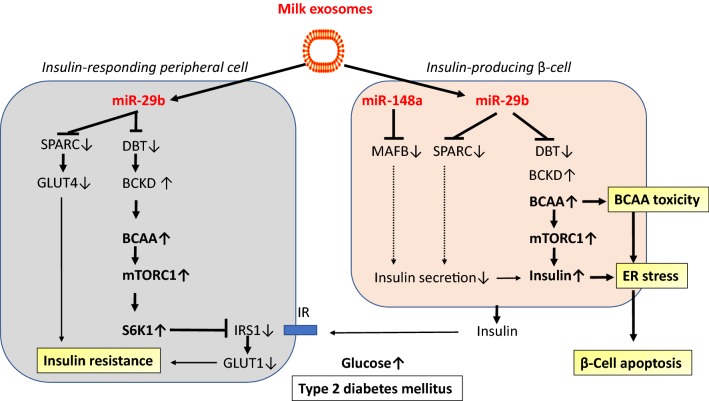
Dairy milk exosomes and type 2 diabetes mellitus. In peripheral tissues (muscle and adipose tissue), miR-29b promotes insulin resistance via inhibiting the core protein *dihydrolipoamide branched*-*chain acyltransferase* (DBT) of *branched*-*chain alpha*-*ketoacid dehydrogenase* (BCKD), the rate limiting enzyme of branched-chain amino acid (BCAA) degradation. Resulting increases in intracellular BCAA levels enhance mTORC1-S6K1 activity. Overstimulated S6K1 phosphorylates and inhibits insulin-receptor substrate 1 (IRS-1) thereby decreasing insulin signaling and glucose transporter 1 (GLUT1) translocation to the cell membrane. In addition, miR-29b-mediated suppression of *secreted protein acidic and rich in cysteine* (SPARC) reduces GLUT4 activity. miR-29b-mediated attenuation of BCAA degradation increases BCAA-mTORC1-mediated insulin synthesis. miR-29b-mediated suppression of SPARC reduces insulin secretion. In addition, miR-148a-mediated suppression of *V*-*Maf musculoaponeurotic fibrosarcoma oncogene homolog B* (MAFB) in the β-cell reduces glucose-dependent insulin secretion. Continued miR-29b-mediated overstimulation of insulin synthesis and miR148a- and miR-29b-mediated impairment of insulin secretion enhances endoplasmic reticulum (ER) stress promoting β-cell apoptosis, the hallmark of type 2 diabetes mellitus

Milk miR-29b-mediated increases in BCAA levels and BCAA-driven mTORC1 activation in peripheral tissues explain insulin resistance by S6K1-mediated inhibitory phosphorylation of insulin receptor substrate 1 (IRS-1), a key checkpoint of insulin signaling [178–181] (Fig. 6). SPARC (*secreted protein acidic and rich in cysteine*, also known as osteonectin or BM-40) may represent an important link between obesity and T2DM [182]. Overexpression of SPARC in cultured β-cells resulted in a 2.4-fold increase in insulin secretion in high glucose conditions [183]. Reduced SPARC expression was demonstrated in primary islets from subjects with diabetes compared with controls [183]. It has been demonstrated that SPARC is a direct target of miR-29b [184, 185]. Importantly, overexpression of miR-29s reduced glucose uptake and GLUT4 levels [185].

The Maf basic leucine-zipper-containing transcription factor MAFB is required for the generation of functional β-cell populations by directly activating insulin gene transcription and key regulators of β-cell differentiation and function [186]. Importantly, MAFB increases the expression of MAFA, which is important to maintain β-cell function in adults [187]. Notably, MAFB is a direct target of miR-148a [188]. Suppressed expression of MAFB in murine and human β-cells has been associated with decreased glucose-dependent insulin secretion [189].

Thus, persistent milk exosome miR-29b-BCAA-mediated overstimulation of insulin synthesis and BCAA-mTORC1-dependent insulin resistance identify dairy milk exosomes as potential promoters of T2DM. Both, miR-29b-mediated suppression of SPARC and miR-148a-mediated suppression of MAFB impair insulin secretion, a potential mechanism enhancing ER stress and β-cell apoptosis.

## Atherosclerosis, cardiovascular and overall mortality

Atherosclerosis and cardiovascular disease are the major causes of death in industrialized countries. Two large Swedish cohorts, one with 61,433 women and one with 45,339 men determined the association between milk consumption and time to mortality [190]. For every glass of milk, which in Sweden is primarily pasteurized milk, the adjusted hazard ratio of all-cause mortality was 1.15 (1.13–1.17) in women and 1.03 (1.01–1.04) in men, respectively. A recent study from Northern Sweden including 103,256 adult participants reported that high consumers of nonfermented milk (≥ 2.5 times/day) had a 32% increased hazard (HR: 1.32; 95% CI 1.18, 1.48) for all-cause mortality compared with that of subjects who consumed milk ≤ 1 time/week [191]. In contrast, fermented milk intake and cheese intake were negatively associated with mortality [191]. A systematic review and updated dose–response meta-analysis of prospective cohort studies partially funded by dairy associations reported a 7% lower risk of stroke with an increment of 200 g milk daily [192]. The association of milk with total stroke was nonlinear, with the strongest inverse association around 125 g/day. For milk intake in the range of 125–750 g/day the inverse association remained significant, but was attenuated. Based on the same studies, whole milk intake was significantly associated with a higher risk of stroke per 200 g/day with no heterogeneity. In contrast, total fermented dairy intake (200 g/day) was associated with a 9% lower risk of stroke [192]. The *Prospective Urban Rural Epidemiology* (PURE) study investigators reported no increase in cardiovascular mortality by dairy intake including milk consumption in low-income and middle-income countries. Milk intake > 1 serving vs no intake was associated with lower risk of cardiovascular mortality [193]. The category >* 1 serving vs no intake* is not suitable to identify a dose-relationship between milk intake and mortality risk. Yogurt was associated with lower risk of cardiovascular mortality [193]. Again, all these questionnaire-based studies did not provide information on the type of thermal processing (pasteurized vs UHT) of milk.

Whereas whole milk consumption appears to be associated with an increased risk of mortality, fermented milk and fermented milk products are not. The presence of milk-derived bioactive exosomes and their miR content may play a key role explaining this discrepancy. Non-coding RNAs and miRs are in the recent focus of lipid and atherosclerosis research [194, 195]. During atherosclerosis, the gradual accumulation of lipids into the subendothelial space of damaged arteries results in several lipid modification processes followed by macrophage uptake in the arterial wall. Cholesterol accumulation within monocyte-derived macrophages and their transformation into foam cells make up the characteristic fatty streaks observed in the early stages of atherosclerosis [196, 197]. Notably, milk-derived exosomes and their miR content are taken up by human macrophages [47]. Exosome-derived miRs are regarded as potential biomarkers of atherosclerosis [198, 199]. It has recently been demonstrated that miR-148a promotes the differentiation of monocytes into macrophages and induces M1 but inhibits M2 polarization [200]. Macrophages overexpressing miR-148a exhibited enhanced ability to engulf and kill bacteria, which was mediated by excessive production of reactive oxygen species (ROS). Furthermore, PTEN has been detected as a direct target gene of miR-148a in macrophages. Macrophages overexpressing miR-148a via upregulation of AKT signaling increased the production of ROS and pro-inflammatory cytokines through upregulation of NF-κB signaling [200]. Peritoneal macrophages of organic dust-exposed mice which were fed a milk exosome-enriched diet exhibited an M1 shift compared to an M2 phenotype in mice fed a milk exosome-deficient diet [201]. In macrophages of mice which received a diet enriched in milk exosomes, interleukin 6 (IL-6), TNF, and IL-12/23 were significantly elevated [201]. Remarkably, a dose-dependent correlation between milk consumption and plasma levels of IL-6 has been reported in humans [190].

**Fig. 7:**
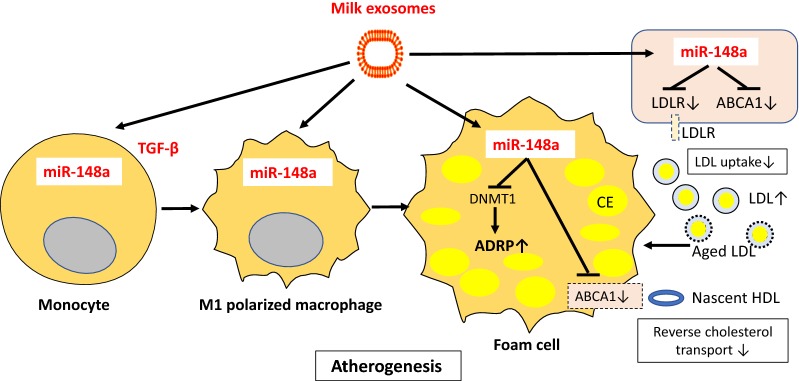
Dairy milk exosomes and atherogenesis. Milk exosomes are taken up by monocytes and macrophages. miR-148a stimulates the differentiation of monocytes to macrophages, especially of macrophages of the pro-inflammatory M1 type. miR-148a-mediated suppression of *low density*-*lipoprotein* (LDL*) receptor* (LDLR) expression increases circulating LDLs that after ageing-dependent chemical modifications are scavenged by macrophages. miR-148a-mediated suppression of *ATP binding cassette transporter 1* (ABCA1) attenuates reverse cholesterol transport and thus further promotes lipid accumulation in macrophages. miR-148a-mediated suppression of DNA methyltransferase 1 (DNMT1) enhances the expression of *adipose differentiation*-*related protein* (ADRP) further promoting foam cell formation

miR-148a directly targets the expression of *low density*-*lipoprotein (LDL) receptors* (*LDLR*) [202, 203], the pivotal regulators of cholesterol homeostasis and hepatic LDL clearance [204]. In addition, miR-148a directly targets *ATP*-*binding cassette transporter 1* (*ABCA1*) [202], the key player for reverse cholesterol transport [205, 206]. For the growing infant, milk exosome-derived miR-148a via suppression of LDL-mediated hepatic cholesterol uptake and impairment of reverse cholesterol transport from peripheral tissues may serve to provide sufficient amounts of cholesterol for growth of distant tissues as well as steroid hormone biosynthesis. The persistence of this lipid metabolic switch into adulthood may however exert atherogenic effects. ABCA1 expression is induced during differentiation of human monocytes into macrophages in vitro [205]. In macrophages, both ABCA1 mRNA and protein expression are upregulated in the presence of acetylated low-density lipoprotein (AcLDL) [205, 206]. Milk exosome-derived miR-148a via targeting ABCA1 may thus attenuate macrophage cholesterol efflux promoting foam cell formation (Fig. 7). In fact, histopathologic examination of ABCA1(−/−) mice at ages 7, 12 and 18 months demonstrated a striking accumulation of lipid-laden macrophages [207]. It has recently been demonstrated that when miR-148a/152 was overexpressed, DNMT1 expression was suppressed, whereas the expression of *adipose differentiation*-*related protein* (ADRP) was enhanced, and the contents of total cholesterol (TC) and cholesteryl ester (CE) were increased in cultured macrophage foam cells [208]. Conversely, downregulation of miR-148a/152 led to elevated DNMT1 expression, reduced ADRP expression, and lowered contents of TC and CE [208]. Antisense miR-148a administration has recently been proposed as a new treatment option of atherogenic dyslipidemia [203].

Several findings connect miRs to cardiovascular pathology. Neointimal formation is a common pathological phenotype in diverse cardiovascular diseases such as atherosclerosis and coronary heart disease. miR-21 has been related to vascular neointimal lesion formation, whereas downregulation of overexpressed miR-21 decreased neointima formation in rat carotid artery after angioplasty [209]. Upregulated miR-21 in endothelial cells suppressed apoptosis and increased eNOS phosphorylation and nitric oxide production [210]. In all these settings, miR-21 upregulation inhibited apoptosis and induced proliferation of vascular smooth muscle cells, contributing to the formation of neointima thickening in vivo [211]. Importantly, it has been demonstrated that milk exosomes are taken up by human vascular endothelial cells via endocytosis [36]. It is thus conceivable that systemic uptake of milk exosomes exerts adverse miR-21-mediated effects on vascular homeostasis.

Taken together, persistent dietary exposure to exosomal miR-148a and miR-21 derived from pasteurized, unfermented milk could exert atherogenic activities that may increase cardiovascular morbidity and mortality.

## Bone remodeling, osteoporosis and fracture risk

It is common belief that consumption of cow’s milk, an abundant source of calcium, promotes bone strength and bone health, a major reason to recommend higher milk intake during pregnancy, infancy, adolescence and adulthood. However, greater milk consumption during teenage years was not associated with a lower risk of hip fracture in older adults [212]. Michaëlsson et al. [190] in Sweden reported that for every glass of milk in women no reduction was observed in fracture risk with higher milk consumption for any fracture or for hip fracture. However, high milk intake was associated with higher fracture incidence in Swedish women [190]. In contrast, among US men and women, 1 glass of milk per day was associated with an 8% lower risk of hip fracture [213]. Thus, the role of dairy foods and the quantity of milk intake for hip fracture still remains controversial. Recent evidence indicates that fermented milk in comparison to non-fermented milk exerts a protective effect on hip fracture rates and bone mineral density [214, 215]. Biver et al. [216] prospectively followed a cohort of 65-year-old healthy Swiss women and showed that age-related Ct bone loss was attenuated at non-bearing bone sites in fermented dairy product consumers, but not in milk consumers, independently of total energy, calcium, and protein intakes. According to a recent study, there was insufficient evidence to deduce the association between milk consumption and risk of hip fracture, which was however reduced by yogurt and cheese consumption [217].

Until today, no epidemiological study considered the heat processing (pasteurization vs UHT) of milk and did not report on the presence or absence of bioactive milk exosomes and their miR cargo [190, 212–225]. The presence of bioavailable milk exosomes is however of utmost importance to understand the differences in the biological function of pasteurized versus UHT milk on bone homeostasis.

Bone structure and homeostasis is controlled by MSCs. In the bone marrow, multipotent MSCs undergo differentiation into various anchorage-dependent cell types, including osteoblasts and adipocytes. At the cellular level, the MSC pool in the bone marrow niche shows a biased differentiation towards adipogenesis at the cost of osteogenesis [226]. This differentiation shift leads to decreased bone formation, contributing to the etiology of osteoporosis [226]. Since the identification of the v-MAF oncogene in an avian tumor virus, the MAF protein family has grown rapidly, forming a unique subclass of basic-leucine zipper transcription (bZIP) factors. MAF family members appear to play important roles in the regulation of MSC differentiation [227]. Nishikawa et al. [228] demonstrated that decreased expression of MAF in mouse MSCs, which regulated MSC bifurcation into osteoblasts and adipocytes by cooperating with the osteogenic transcription factor RUNX2 and inhibiting the expression of the adipogenic transcription factor PPARγ, impaired osteogenesis [228]. The crucial role of MAF in both osteogenesis and adipogenesis was underscored by in vivo observations of delayed bone formation in perinatal MAF(−/−) mice, and accelerated formation of fatty marrow associated with bone loss in aged MAF(±) mice.

**Fig. 8:**
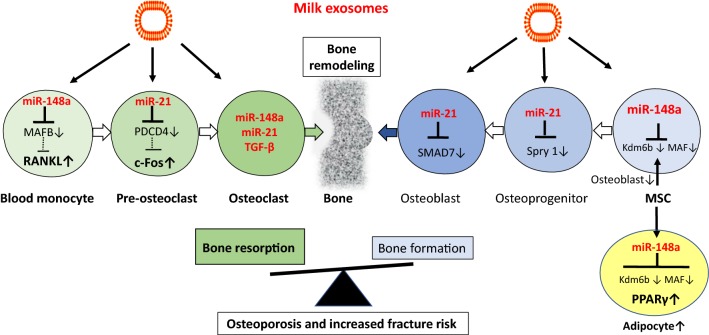
Milk exosome and bone homeostasis. MiRs play a key role in the regulation of bone remodeling executed by bone-resorbing osteoclasts and bone-forming osteoblasts. Blood monocytes are a primary source of osteoclast precursor cells. Upregulation of *receptor activator of nuclear factor B ligand* (RANKL), *V*-*Fos FBJ murine osteosarcoma viral oncogene homolog* (c-Fos) and *transforming growth factor*-*β* (TGF-β) promote osteoclastogenesis. miR-148a via targeting *V*-*maf musculoaponeurotic fibrosarcoma oncogene homolog B* (MAFB) increases RANKL expression. miR-21 via targeting *programmed cell death 4* (PDCD4) increases c-Fos activity. Notably, miR-148a, miR-21 and TGF-β are provided by dairy milk exosomes. Addition of commercial milk-derived exosomes to bone marrow-derived osteoclast precursor cells increased osteoclast formation. Overexpression of miR-148a triggers mesenchymal stem cells (MCS) to differentiate into adipocytes and attenuates osteoblast differentiation. Persistent intake of dairy milk exosomes may thus disturb the delicate balance of bone remodeling favoring osteoclastogenesis over osteoblastogenesis, a critical mechanism promoting osteoporosis and fracture risk

MAF and MAFB are direct target genes of miR-148a [188, TargetScanHuman7.2]. Long-term exposure to milk exosome-derived miR-148a may thus favor adipogenesis in the bone on the expense of osteogenesis. Bone remodeling is a life-long process to maintain bone homeostasis. Its imbalance causes bone porosity and increases the risk of fracture. The balance is controlled by bone-forming osteoblasts and bone-resorbing osteoclasts interacting with blood-vessel-forming endothelial cells [229]. There is compelling evidence that exosomes and their miR cargo play a crucial role in bone remodeling [230–236]. Kelch et al. [234] recently reported that miR-148a and miR-21 are significantly upregulated in serum and osteoclasts of patients with osteoporosis. In accordance, increased levels of miR-148a and miR-21 have been detected in sera of type 1 diabetes patients (T1DM) versus non-diabetic subjects. In patients with T1DM, who exhibit reduced bone mineral density (BMD) associated with an increased risk of fractures, miR-148a expression showed an inverse correlation with BMD [237]. Remember that milk exosomal miRs have been shown to increase in PBMCs of milk consumers [46]. In addition, the uptake of dairy milk exosomes and their miRs by human macrophages has also been demonstrated [47]. Peripheral blood monocytes (PBMs) are an important source of osteoclast precursors and cytokines produced by PBMs have profound effects on osteoclast differentiation, activation, and apoptosis [238]. *Receptor activator of nuclear factor kappaB ligand* (RANKL) induces osteoclast formation from hematopoietic cells via regulation of various transcription factors. MAFB negatively regulates RANKL-induced osteoclast differentiation [239]. Intriguingly, miR-148a targets MAFB, a critical inhibitor of RANKL, thereby promoting the differentiation of monocytes to pre-osteoclasts [188]. miR-148a was reported to be dramatically upregulated during M-CSF + RANKL-induced osteoclastogenesis of CD14^+^ PBMCs [188]. miR-21, another exosomal signature miR of dairy milk, suppresses *programmed cell death 4* (PDCD4) [240, 241], a critical inhibitor of c-Fos, which is important for the differentiation of pre-osteoclasts to osteoclasts [234]. Intriguingly, Oliveira et al. [232], demonstrated that dairy milk exosomes (100,000×*g* fraction) promoted osteoclast differentiation associated with an increased expression of c-Fos (Fig. 8).

Oral administration of dairy milk-derived exosomes to female DBA/1 J mice during 7 weeks did not alter the tibia trabecular bone area but increased the number of osteocytes [242]. The highest dose of milk exosomes markedly increased the woven bone tissue. The exposure of MSCs to bovine milk exosomes during 21 days resulted in less mineralization but higher cell proliferation. Interestingly, milk exosomes reduced the collagen production, but enhanced the expression of genes characteristic for immature osteoblasts [242]. A kinetic study showed that milk exosomes upregulated many osteogenic genes within the first 4 days. However, the production of type I collagen and expression of its genes (COL1A1 and COL1A2) were markedly reduced at days 21 and 28. At day 28, milk exosomes again lead to higher proliferation, but mineralization was significantly increased. This was associated with increased expression of sclerostin, a marker for osteocytes, and reduced SPARC (osteonectin), which is associated to bone matrix formation and bone mineralization [242].

Earlier studies suggested that TGF-β increases osteoclast formation via action on osteoclast precursors [243]. TGF-β is a component of dairy milk exosomes [14] and has been shown to promote the differentiation of blood monocytes into osteoclasts [240]. Addition of TGF-β and dexamethasone to peripheral blood (PB) monocytes led to higher number of nuclei in multinuclear cells and increased expression of tartrate resistant acid phosphatase (TRACP) 5a and 5b, CR and NFATc1 in PB-derived osteoclasts depicting the higher osteoclastogenic potential and responsiveness to TGF-β and dexamethasone in PB monocytes [244].

There is further evidence that miR-148a inversely regulates adipocyte and osteoblast differentiation [233]. Supplementing miR-148a activity inhibited cell growth and induced stromal ST2 cells to differentiate into mature adipocytes. By contrast, supplementation of miR-148a blunted osteoblast differentiation. Lysine-specific demethylase 6b (Kdm6b), a recently identified regulator of osteoblast differentiation, was shown to be a direct target of miR-148a. Overexpression of Kdm6b attenuated miR-148a-mediated stimulation of adipogenic differentiation. Thus, miR-148a reciprocally regulates adipocyte and osteoblast differentiation through directly targeting Kdm6b [233].

Collectively, accumulating translational evidence supports the view that persistent uptake of pasteurized dairy milk and their bioactive exosomal miRs after the skeletal growth period activate osteoclastogenesis and impairs osteoblastogenesis, an unfavorable deviation for adults disturbing the appropriate balance for bone remodeling and explaining the association of obesity, diabetes mellitus and osteoporosis [245] (Fig. 8).

## Parkinson’s disease

Epidemiological evidence supports a correlation between milk intake and risk of Parkinson’s disease (PD) [246–251]. A large meta-analysis reported a linear dose–response relationship for milk consumption and PD [249]. PD risk increased by 17% for every 200 g/day increment in milk intake [249]. Analyses were based on data from 2 large prospective cohort studies, the *Nurses’ Health Study* (n = 80,736) and the *Health Professionals Follow*-*up Study* (n = 48,610) confirmed an increased risk of PD associated with consumption of skim and low-fat milk [251]. Notably, there is no increased PD risk for fermented milk products such as yogurt [248, 249]. Neuron density in *substantia nigra* was lowest in nonsmoking decedents who consumed high amounts of milk (> 473 ml/day) [250]. After removing cases of PD and dementia with Lewy bodies, adjusted neuron density in all but the dorsomedial quadrant was 41.5% lower for milk intake > 473 ml/day versus intake that was less [250]. Thus, milk intake, but not fermented milk appears to exert neurodegenerative effects in PD.

Growing evidence indicates that exosomes are prominent mediators of neurodegenerative diseases. Exosomes of PD patients contain neurodegenerative disease-associated proteins such α-synuclein (α-syn) and facilitate their spread to the extracellular environment [252–255]. There is increasing evidence that exosome lipids promote α-syn aggregation [256]. Aggregation of exogenous α-syn was accelerated by exosomes irrespective of whether they were derived from control cells or cells overexpressing α-syn suggesting that the lipids in exosomes were sufficient for the catalytic effect to arise [252, 256]. As milk exosomes have been detected to cross the blood–brain barrier and accumulate in the brain [39], it is conceivable that dairy milk exosomes may promote α-syn aggregation and spreading.

There is recent interest in the regulatory role of exosomal miRs in the pathogenesis of PD [257], which according to recent concepts is related to neuroinflammation [258]. Prajapati et al. [259] demonstrated that TNFα is a potential regulator of miRs which may regulate mitochondrial functions and neuronal cell death, having important implication in pathogenesis of PD. TNFα induced the expression of miR-155 [259]. Recently, Thome et al. [260] found significant upregulation of miR-155 in an in vivo model of PD produced by adeno-associated-virus-mediated expression of α-syn. Using a mouse with a complete deletion of miR-155, they found that a loss of miR-155 reduced proinflammatory responses to α-syn and blocked α-syn-induced neurodegeneration. In primary microglia from miR-155(−/−) mice, they observed a markedly reduced inflammatory response to α-syn fibrils. Treatment of these microglia with a synthetic mimic of miR-155 restored the inflammatory response to α-syn fibrils. These results suggest that miR-155 plays a central role in the inflammatory response to α-syn in the brain and in α-syn-related neurodegeneration [260]. Importantly, miR-155 is one of the major immune regulatory miRs in cow’s milk that most likely invades into the brain [39].

**Fig. 9:**
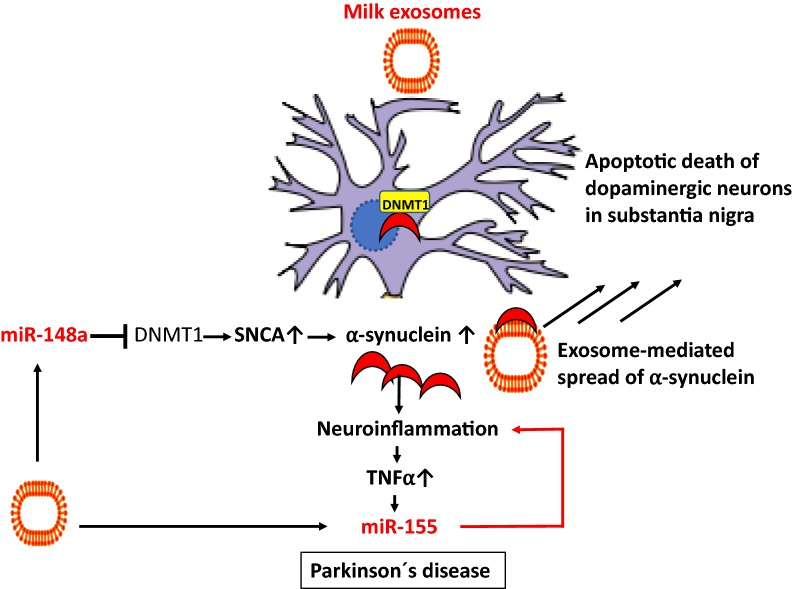
Dairy milk exosomes and pathogenesis of Parkinson’s disease. Milk exosomes preferentially accumulate in the brain. Milk exosome-derived suppression of DNA methyltransferase 1 (DNMT1) reduces *SNCA* promoter methylation resulting in increased expression of α-synuclein. α-Synuclein promotes nuclear extrusion of DNMT1. Aggregates of α-synuclein induce neuroinflammation and increase tumor necrosis factor-α (TNF-α)-mediated upregulation of miR-155, which further enhances neuroinflammation. Milk exosomes via binding of α-synuclein to exosome membrane lipids may promote the spread of neurotoxic α-synuclein in the brain. Suppression of miR-155 attenuated α-synuclein-induced neuroinflammation in models of Parkinson’s disease

Methylation of human α-syn gene *SNCA* intron 1 decreased its gene expression, while inhibition of DNA methylation activated SNCA expression. Methylation of *SNCA* intron 1 was reduced in DNA from sporadic PD patients’ substantia nigra, putamen, and cortex [261]. In fact, CpG demethylation in the promoter region of SNCA enhances α-syn expression and affects the pathogenesis of PD [262]. It has been shown that α-syn sequesters DNMT1 from the nucleus, which might be a novel mechanism for epigenetic alterations in Lewy body diseases [263]. Milk exosome-derived miR-148a may be another epigenetic mechanism, which via targeting DNMT1 may increase α-syn expression promoting PD pathogenesis [8] (Fig. 9).

Collectively, milk exosomes and their miRs, which accumulate in the brain after milk consumption [39], might be the critical promoters involved in the initiation and progression of PD in humans consuming pasteurized cow’s milk.

## Colorectal cancer

Two large meta-analyses came to the conclusion that milk consumption but not the consumption of fermented milk products has a protective effect on the development of colorectal cancer (CRC) [264, 265]. A recent systematic review and meta-analysis of cohort studies confirmed that an increase of 200 g/day of milk intake was associated with a decreased risk of CRC [266]. It has been shown that bacterial fermentation attacks the integrity of cow milk exosomes associated with a reduction of miR recovery [48, 49]. The protein content and size of bovine milk exosomes was significantly reduced in fermented cow’s milk associated with a substantial loss of miRs (miR-29b, miR-21) compared to unfermented raw milk [48]. Malignant epithelial cells of CRC exhibit a reduced expression of miR-148a [267–270], which increases the expression of DNMT1 that functions as a tumor promoter in CRC [271–273]. Intriguingly, Golan-Gerstl et al. [44] demonstrated that the incubation of CRC cells (Lim 1215) with human breastmilk exosomes increased the cellular content of miR-148a. In exosome incubation experiments with normal intestinal cells (CRL 1831), a significant decrease in DNMT1 was observed [44]. It is thus possible that milk exosome-mediated uptake of bovine miR-148a, which is identical with human miR-148a (mirbase.org), targets DNMT1 and thereby attenuates the action of this critical promoter of CRC initiation and progression. Increased expression of *Rho*-*associated coiled coil*-*containing protein kinase 1* (ROCK1) also plays a key role in CRC pathogenesis [274, 275], which as well is a direct target of miR-148a [276].

Chronic inflammation triggers cellular events that can promote malignant transformation of cells and carcinogenesis. Chronic intestinal inflammation is a well-known stimulus of CRC cancerogenesis [277]. Several inflammatory mediators, especially TNFα, IL-6 and IL-10 have been shown to participate in both the initiation and progression of cancer including colitis-associated CRC [277]. miR-148a has a substantial impact on immune regulation and cancerogenesis [278]. miR-148a inhibits the production of cytokines including TNFα, IL-6, IL-12 and impairs innate response and antigen presentation of Toll-like receptor (TLR)-triggered dendritic cells by targeting calcium/calmodulin-dependent protein kinase IIα (CaMKIIα) [279]. Furthermore, TGF-β and miR-155, both components of milk exosomes, suppressed intestinal T cells and had a protective effect on the development of colitis [84].

**Fig. 10:**
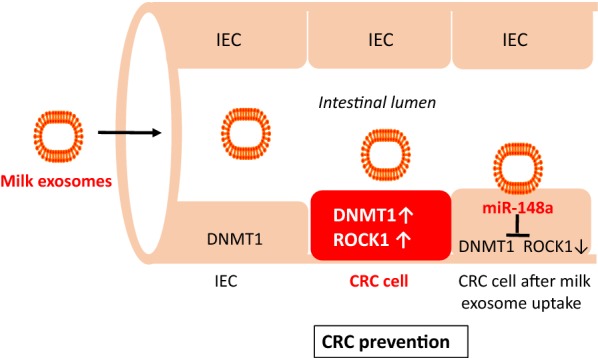
Dairy milk exosomes and colorectal cancer prevention. *DNA methyltransferase 1* (DNMT1) and *Rho*-*associated coiled coil*-*containing protein kinase 1* (ROCK1) are overexpressed in colorectal cancer (CRC) cells. DNMT1 is regarded as a tumor promoter in CRC. Milk exosome uptake by CRC intestinal epithelial cells (IEC) increases intracellular levels of miR-148a, which suppresses DNMT1 and ROCK1, a potential mechanism explaining CRC prevention by consumption of pasteurized milk

Thus, mounting epidemiological and translational evidence indicates that milk exosomes via transfer of miR-148a and miR-155 may have a preventive effect on CRC cancerogenesis (Fig. 10).

## Prostate cancer

Among European men, prostate cancer (PCa) is the most common cancer and the third leading cancer cause of death [280]. In 2018, there were an estimated 3.91 million new cases of cancer including 450,000 cases of PCa [280]. In the *Physicians’ Health Study* (n = 21,660) an association between whole milk intake and PCa-specific mortality among U.S. male physicians has been demonstrated [149]. A large meta-analysis of 11 population-based cohort studies involving 778,929 individuals reported a linear dose–response relationship between increase of whole milk intake and increase of PCa mortality risk [281]. A recent study confirmed that in comparison to men who consumed < 1 servings/day of whole milk, those who drank ≥ 3 servings/day had an increased hazard of PCa mortality [282]. In 996 African American and 1064 European American men diagnosed with PCa, a higher whole milk intake was associated with higher odds of high-aggressive PCa [283]. Pettersson et al. [284] showed that men with the highest *versus* lowest intake of whole milk were at an increased risk of PCa progression. A prospective study among 1334 men with non-metastatic PCa in the *Cancer of the Prostate Strategic Urologic Research Endeavor (*CaPSURE™) reported that whole milk consumption after PCa diagnosis was associated with increased risk of recurrence, particularly among very overweight or obese men [285]. Milk fat contains branched-chain fatty acids, whose metabolism is disturbed in PCa patients. A central role for fatty acid oxidation in supplying energy to the PCa cell is supported by the observation that the peroxisomal enzyme α-methylacyl-CoA racemase (AMACR), which facilitates the transformation of branched-chain fatty acids to a form suitable for β-oxidation, is highly overexpressed in PCa compared with normal prostate [286–288]. Branched-chain fatty acids in milk and dairy products markedly enhance AMACR expression in PCa cells in vitro [289].

In contrast to whole milk, total dairy and fermented milk products did not correlate with PCa risk [281–285]. Milk fat and calcium are obviously not the primary causative nutritional factors in whole milk for PCa initiation and progression, as milk fat is abundant in cheese and other fermented milk products. In contrast to fermented milk products, pasteurized milk transfers milk exosomes to the consumer.

Cancer cells communicate closely with the cells in their microenvironment, and this communication promotes malignancy via abnormal growth, invasion, drug resistance and metastasis. Increasing evidence illustrates that exosomes derived from tumor cells trigger tumor initiation, tumor cell growth and progression, metastasis, and drug resistance [290–297]. Exosome release by PCa cells modify the tumor microenviroment and play a key role in PCa initiation and progression [298–303].

miR-21, a signature miR of dairy milk, is overexpressed in PCa cells [304, 305], in blood serum [304, 306], PBMCs [307], serum exosomes [308] well as urinary exosomes of PCa patients [309, 310]. miR-21 is regarded as an oncomir, that inhibits pivotal tumor suppressor genes such as PTEN, p57kip2 (CDKN1C), PDCD4, MARCKS and others [240, 311–314]. Loss of function of the PTEN tumor suppressor, upregulating the phosphoinositide 3-kinase (PI3K)-AKT signaling network, is recognized as one of the most common driving events in PCa development [311]. Overexpression of miR-21 has been associated with chemo resistance and PCa progression [315, 316]. In accordance, an increase in miR-21 helps PCa cells to overcome androgen deprivation [317].

Epithelial–mesenchymal transition (EMT) plays a pivotal role in the conversion from benign to malignant phenotypes. There is accumulating evidence that exosomes via miR transfer prepare the pre-metastatic niche [318]. TGF-β signaling plays a further key role in EMT-mediated cancer progression [319]. PCa-derived exosomes, which in accordance with milk exosomes contain TGF-β [14], dominantly dictated a program of MSC differentiation generating myofibroblasts with functional properties consistent with cancer promotion [320]. Remarkably, it has been shown that human breast milk exosomes could promote EMT via TGF-β2 [15]. Commercial milk exosomes via transfer of TGF-β may further augment TGF-β-mediated EMT. Recent evidence has been provided that inhibition of DNMT1 induces EMT and the cancer stem cell (CSC) phenotype facilitating tumorigenesis in PCa cells [321]. Notably, miR-148a, miR-21 and miR-29b, major miRs of dairy milk exosomes, synergistically attenuate DNMT1 expression [68–71]. Furthermore, DNMT1 negatively controls the activity of androgen receptor signaling, which plays a key role in PCa pathogenesis [322].

miR-155 is upregulated in PCa tissues and cell lines and promotes cell proliferation by targeting annexin 7 [323]. SOCS1 functions as a tumor suppressor in PCa and its expression is reduced in PCa tissue [324]. miR-221-mediated suppression of SOCS1 enhanced cell proliferation and metastasis through in PCa [325]. Importantly, miR-155 as well targets SOCS1 [77]. miR-155 is an important exosomal immune regulatory miR of human and bovine milk [17, 45, 84]. miR-30d is another suppressor of SOCS1 [326]. miR-30d is overexpressed in PCa tissue and is inversely related to SOCS1 expression [326]. Remarkably, miR-30d is another signature miR of commercial cow’s milk [84].

Milk consumption has been linked with increased expression of *fat mass and obesity*-*associated gene* (FTO) via miR-148a-mediated suppression of DNMT1 [327]. Epidemiology studies show that FTO SNPs (including rs9939609, rs17817449, rs8050136, rs1477196, rs6499640, rs16953002, rs11075995, and rs1121980) are associated with increased FTO expression, overweight/obesity and increased risk of various types of cancers, including PCa [328]. FTO has recently been shown to increase the expression of C/EBPα and C/EBPβ [329, 330], which are upregulated in PCa tissue [331–333]. Remarkably, a C/EBP binding motif has been identified in the *AMACR* promotor [334]. Milk exosome-derived miR-148a via epigenetic enhancement of FTO-C/EBP-signaling may enhance AMACR expression in PCa allowing malignant cells to utilize branched-chain fatty acids as an alternative energy source for PCa growth and metastasis.

Bernichtein et al. [335] failed to observe any proliferative effects of “whole cow’s milk” in two mouse models of benign prostatic hyperplasia (probasin-Prl mice, Pb-Prl) or pre-cancerous PIN lesions (KIMAP mice). They reported decreased levels of the cell proliferation marker Ki-67. Notably, these investigators did not use “whole milk” as claimed on their paper’s title but instead used powdered milk re-suspended in water [335]. There is good reason to assume that cow milk powder in analogy to infant formula misses bioactive exosomal miRs [86]. In contrast, Tate et al. [336] observed a 30% increase in proliferation of LNCaP cells in culture after addition of commercial cow’s milk, which contains bioactive milk exosomes. The incidence of lactose intolerance, a natural protection for milk and milk exosome consumption, in PCa patients was significantly less than that in the control group [337].

**Fig. 11:**
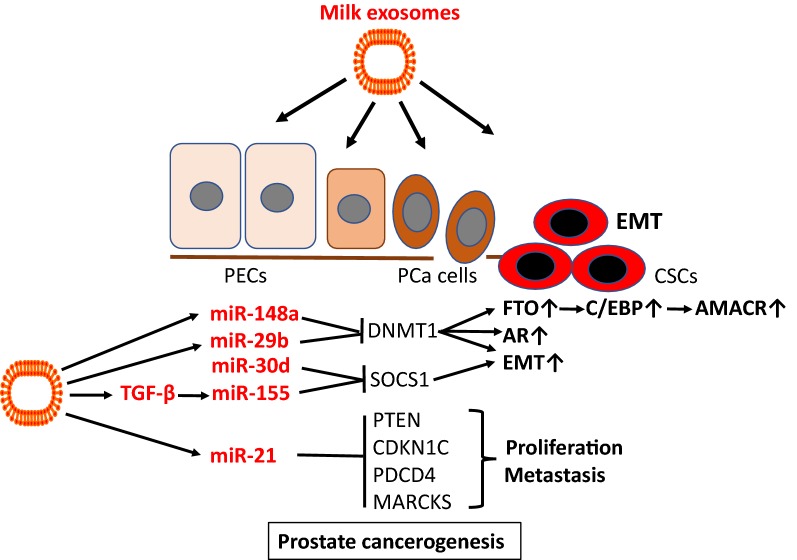
Dairy milk exosomes and prostate tumorigenesis. Milk exosome-derived miR-148a, miR-29b and miR-21 suppress DNA methyltransferase 1 (DNMT1), a critical step in prostate cancer (PCa) epithelial–mesenchymal transition (EMT) and cancer stem cell (CSC) formation. miR-148a-mediated suppression of DNMT1 enhances the expression of *fat mass and obesity*-*associated gene* (FTO), which increases the expression of *CCAAT enhancer element binding protein*-*β* (C/EBPβ), a potential mechanism increasing the expression of *α*-*methylacyl*-*CoA racemase* (AMACR). DNMT1 is also a negative regulator of androgen receptor (AR) signaling. Milk exosomes provide miR-155 and transforming growth factor-β (TGFβ), which further induces miR-155, which inhibits *suppressor of cytokine signaling 1* (SOCS1), a pivotal inhibitor of EMT. Milk-derived exosomal miR-21 increases the pool of a key oncogenic miR, which suppresses key checkpoint regulators of cell cycle progression and apoptotic signaling including *phosphatase and tensin homolog* (PTEN), *cyclin*-*dependent kinase inhibitor 1C* (CDKN1C, p57kip2), *programmed cell death 4* (PDCD4), *myristolylated alanine*-*rich protein kinase C substrate* (MARCKS) and others

Dairy milk exosomes via transfer of oncogenic miRs and TGF-β may promote growth and PCa progression in consumers of pasteurized whole milk but not fermented milk or milk protein preparations (Fig. 11).

## Breast cancer

Breast cancer (BC) is the most common cancer in women in industrialized countries. In Europe, 523,000 cases of female BC have been estimated in 2018 [280]. A prospective study of 25,892 Norwegian women reported that consumers of 750 ml or more of full-fat milk daily had a relative risk of 2.91 compared with those who consumed < 150 ml [338]. Wang et al. [339] surveyed risk factors for BC in women (n = 122,058) residing in urban and rural areas of eastern China. Among women residing in rural areas, obesity and a high intake of milk were identified as risk factors for BC. A case–control study in Mexico (97 BC patients, 104 controls) reported that high milk consumption increased BC risk by 7.2 times, whereas the consumption of meat was not significantly associated with BC risk [340]. According to a case–control study (n = 333) in Uruguay, high intakes of whole milk was associated with significant increased risk of BC, whereas fermented milk products were associated with significant decreased risk [341]. In a large Swedish cohort (n = 22,788), people with lactose intolerance, characterized by low consumption of milk, had decreased risks of BC [342]. In contrast, an older pooled analysis of cohort studies (n = 351,041) [343] found no significant associations between intake of dairy products and risk of BC. Notably, at present no epidemiological study clearly compared the effect of whole milk versus fermented milk products and there are missing data on the type of heat processing of milk in all epidemiological studies.

Although, epidemiological correlations for whole milk consumption and BC are less established than those for whole milk intake and PCa, tumor-derived exosomes as well play a key role in tumor initiation and progression in BC [344–349]. The widespread post-transcriptional regulatory role of miRs is of recent interest in estrogen receptor (ER)-positive BC, comprising about 65%–70% of BCs [350]. Estrogen/ERα activation can modulate miR expression, which may contribute to ER+ breast carcinogenesis [350]. Estradiol (E2) treatment of BC MCF7 cells doubled the expression levels of miR-148a and miR-21 [351]. An ER binding site has been demonstrated on the *MIR21* gene [352]. E2 induced miR-148a in MCF-7 and MDA-MB-231 cells [353]. miR-21 is overexpressed in BC compared with normal breast tissue and has been associated with advanced stage, lymph node positivity, and reduced survival time [354, 355]. miR-21 is a major miR component of exosomes released by cancer-associated fibroblasts and cancer-associated adipocytes [356, 357], which promote tumor progression [358, 359]. In BC patients, increased miR-21 in the systemic circulation exists either freely or in exosomes [360]. Notably, in postmenopausal women 6 weeks of tamoxifen treatment decreased miR-21 levels suggesting that this miR may be important for BC tumorigenesis [361]. Circulating levels of miR-21 are significantly higher in plasma samples of BC patients, when compared healthy controls [360, 361]. miR-21 is even regarded as a marker of BC exosomes [362] and was found to be selectively enriched in human BC exosomes in the plasma of patients with BC [362–364]. A further increase of exosomal miR-21 via milk intake is apparently not suitable for patients with BC, neither the transfer of milk-derived exosomal miR-148a. In BC cell line MCF7, a miR-148a mimic increased estrogen receptor-α (ERα) expression, whereas a miR-148a inhibitor decreased ERα expression [365]. It has been shown that miR-148a regulates ERα expression through DNMT1-mediated DNA methylation in BC cells [366]. In contrast, it has been reported that miR-148a targets B-cell lymphoma 2 (BCL-2), which is frequently upregulated in BC [367]. Nuclear receptor NR4A1 (Nur77) promotes BC invasion and metastasis by activating TGF-β signaling [368, 369]. NR4A1 binding induces a BCL-2 conformational change that exposes its BH3 domain, resulting in conversion of BCL-2 from an anti-apoptotic to a pro-apoptotic protein [370].

BRCA1, a well-known tumor suppressor, abrogates the repression of miR-155, a *bona fide* oncomir [371]. miR-155 is overexpressed in BC tissue and accelerates the growth of tumor cell lines in vivo and induces tamoxifen resistance [371, 372]. In BC cells, FoxP3 induced miR-155 through transcriptional repression of BRCA1. Notably, miR-155 is known to induce FoxP3 expression [373]. For patients with early stage or localized BC, there were high levels of miR-155 in both plasma and blood cells [373]. Santos et al. [374] recently reported that exosomes enriched in miR-155 added to BC cells induced chemo resistance and promoted EMT. Ectopic expression of miR-155 significantly promoted the proliferation of BC cells, the formation of soft agar foci in vitro, and the development of tumors in nude mice [374]. In BC cells, RNA interference silencing of SOCS1 recapitulates the oncogenic effects of miR-155, whereas restoration of SOCS1 expression attenuated the tumor-promoting function of miR-155, suggesting that miR-155 exerts its oncogenic role by negatively regulating SOCS1 [375]. Thus, exosomal transfer of miR-155, a miR component of colostrum and mature cow’s milk [86, 376], may promote BC tumorigenesis.

TGF-β signaling features a growth inhibitory effect at an early stage but aggressive oncogenic activity at the advanced malignant state [377–379]. Recent efforts in BC therapy are directed against growth factor pathway including TGF-β signaling in BC [379]. Notably, TGF-β1 has been shown to promote the expression of miR-155 [380]. TGF-β2 is significantly upregulated in breast milk exosomes during weaning/early involution. Breast milk exosomes containing high levels of TGF-β2 induced changes in both benign and malignant breast epithelial cells, consistent with the development and progression of BC, suggesting a role for high TGF-β2-expressing breast milk exosomes in influencing BC risk [14]. BC exosomes contain TGF-β2, which suppresses T cell proliferation, a critical pathway used by BC cells to escape immune surveillance [381]. In addition, exosomes from BC cells via TGF-β upregulation converted adipose tissue-derived MSCs into myofibroblast-like cells [382].

**Fig. 12:**
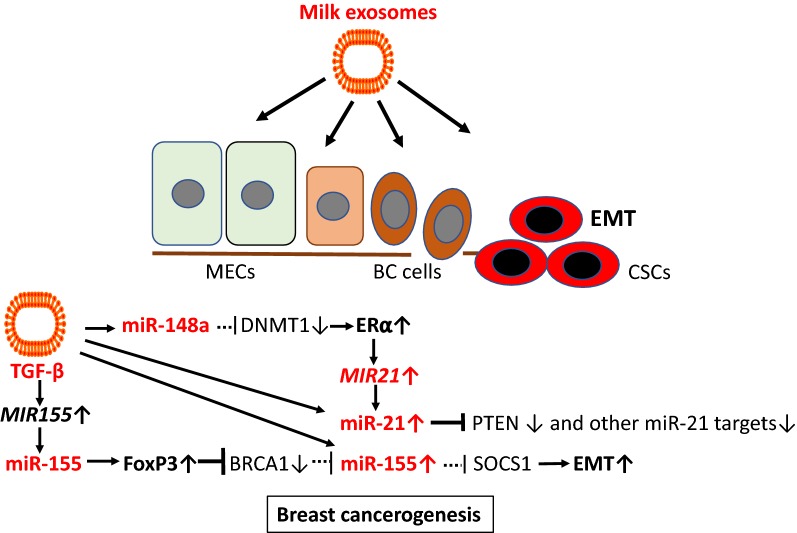
Dairy milk exosomes and breast cancerogenesis. Milk exosomes via transfer of miR-148a and miR-148a-mediated suppression of *DNA methyltransferase 1* (DNMT1) enhances the expression of *estrogen receptor*-*α* (ERα). ERα promotes des expression of miR-21, which targets critical genes involved in PI3K-AKT signaling and cell cycle control. Exosome-derived *transforming growth factor*-*β* (TGF-β) induces the expression of miR-155. miR-155 enhances the expression of FoxP3, a critical inhibitor of the tumor suppressor *breast cancer 1 gene* (BRCA1). Downregulation of BRCA1 further enhances the expression of miR-155, which is a pivotal inhibitor of *suppressor of cytokine signaling 1* (SOCS1) finally promoting epithelial–mesenchymal transition (EMT). Dairy milk exosomes thus contribute to BC tumorigenesis via enhancing key oncogenic components involved in the pathogenesis of BC

Collectively, BC-derived exosomes and dairy milk-derived exosomes both contain and transfer miR-21, miR-155 and TGFβ2, which may exert synergistic effects in breast cancerogenesis (Fig. 12).

## Hepatocellular carcinoma

In the *European Prospective Investigation into Cancer and Nutrition* cohort including 477,206 participants showed a significant positive hepatocellular carcinoma (HCC) risk for the consumption of milk and cheese but not yogurt [383]. Increased expression of miR-148a has been reported in hepatitis B virus-induced HCC resulting from HBx antigen-induced upregulation of *von Willebrand factor C and EGF domain*-*containig protein* [384]. Hepatitis C virus-induced upregulation of miR-155 has been shown to promote hepatocarcinogenesis [385]. miR-21 expression was significantly upregulated in HCC tissues relative to nontumor livers [386]. Exosome-associated miR-21 is markedly elevated in serum of patients with HCC [387]. It has been reported that miR-155 is linked to the recurrence and prognosis of HCC following liver transplantation [381]. In the HCC cell line Huh-7, miR-155 is overexpressed and exhibited altered levels of expression of certain cellular adhesion molecules related to EMT [381]. Furthermore, TGF-β1 upregulated the expression of miR-155 in HCC cells in vitro, which led to the conclusion that increased levels of miR-155 in HCC cells, possibly due to stimulation by TGF-β1, accelerate EMT in the liver. Notably, the liver is a major target of bovine milk exosomes [39]. Recent evidence indicates that miR-155 suppresses *p53*-*induced nuclear protein 1* (TP53INP1), a critical step that is involved in liver cancer stem cell acquisition and self-renewal [388]. TP53INP1 is a p53-inducible gene that regulates p53-dependent apoptosis, downregulates the expression of SPARC and is repressed by miR-155 [389–391]. Recent findings indicate that loss of SOCS1-dependent control over EMT may contribute to MET-mediated migration, invasion and metastatic growth of HCC [392].

**Fig. 13:**
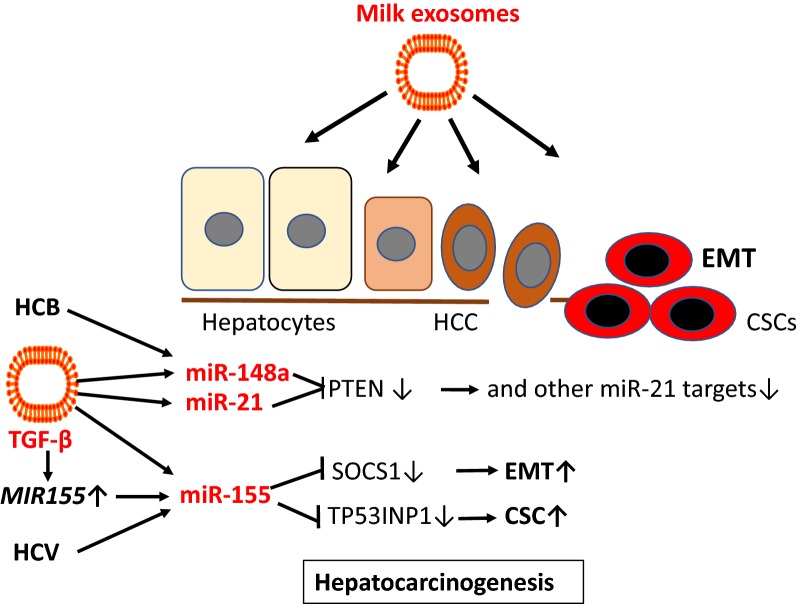
Dairy milk exosomes and hepatocellular carcinoma. After oral administration milk exosomes accumulate preferentially in the liver and may increase the hepatocellular levels of miR-148a, miR-21 and miR-155, which are upregulated in hepatocellular carcinoma (HCC). In hepatitis B virus (HCB)-associated HCC overexpression of miR-148a may be further increased by milk exosome-derived miR-148a. Milk exosome-derived *transforming growth factor*-*β* (TGF-β) may further increase the expression of miR-155, which downregulates *suppressor of cytokine signaling 1* (SOCS1), thereby enhancing epithelial–mesenchymal transition (EMT). MiR-155-mediated suppression of *p53*-*induced nuclear protein 1* (TP53INP1) promotes cancer stem cell (CSC) proliferation in the liver. Milk exosomes may thus increase the risk for HCC. Thus, milk exosomes may augment the tumorigenic effects of hepatitis B (HCB) and hepatitis C virus (HCC)-induced upregulation of miR-148a and miR-155, respectively

Translational evidence indicates that milk-derived exosomes via transfer of onocogenic miR-148a, miR-21, miR-155 and TGF-β may promote the development of HCC (Fig. 13).

## Diffuse large B-cell lymphoma

A recent meta-analysis investigated the relation between dairy product consumption and Non-Hodgkin lymphoma (NHL). Milk has been identified to increase the risk of *diffuse large B*-*cell lymphoma* (DLBCL) [393]. The dose–response analysis suggested that the risk of NHL increased by 6% for each 200 g/day increment of milk consumption but not yogurt [393].

**Fig. 14:**
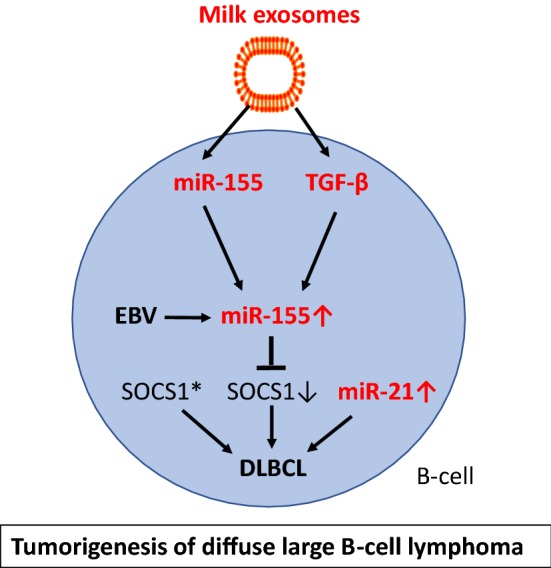
Dairy milk exosomes and tumorigenesis of diffuse large B-cell lymphoma. miR-155 levels are significantly upregulated in diffuse large B-cell lymphoma (DLBCL). Epstein Barr Virus (EBV) infection in DLBCL induces miR-155 expression, which attenuates the expression of *suppressor of cytokine signaling 1* (SOCS1). Loss-of-function mutations of SOCS1 (SOCS1*) have also been found in DLBCL further reducing SOCS1. Milk exosomes via transfer of miR-155 and miR-155-inducing transforming growth factor-β (TGF-β) may further promote tumorigenesis of DLBCL

DLBCL have 10- to 30-fold higher copy numbers of miR-155 than do normal circulating B cells [394]. Epstein Barr virus (EBV)-positive DLBCL is an entity included in the 2016 WHO classification of lymphoid neoplasms [395]. It has been demonstrated that the expression of miR-155 is induced by EBV [396]. EBV acts on noninfected macrophages in the tumor through exosome secretion and thereby augments lymphoma development [397]. Plasma miR-155 expression was significantly upregulated in DLBCL patients compared to healthy individuals [398]. DLBCL cases with an elevated level of miR-155 had shorter overall survival than those with a lower miR-155 expression [398]. Intriguingly, SOCS1, the target of miR-155 and miR-30d, is frequently mutated in patients with DLBCL [399–401]. One-fourth of DLBCL and follicular lymphomas carried SOCS1 mutations, which were preferentially targeted to SHM hotspot motifs and frequently inactivating mutations [401]. Furthermore, increased serum expression levels of miR-21 have been detected in patients with DLBCL associated with negative prognostic outcome [402]. Exosomal transfer of milk-dervived miR-155 and miR-21 to circulating B-cells may initiate or promote DLBCL progression (Fig. 14).

## *MIRNA148A* and dairy cow lactation performance

**Fig. 15:**
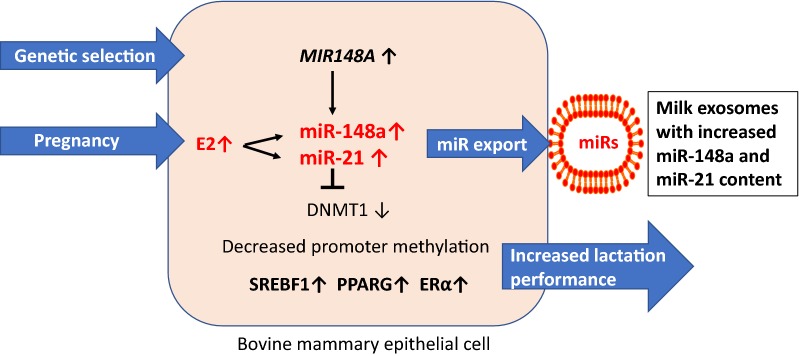
Hypothesized increase in dairy milk exosomal miR content by genetic selection and persistent pregnancy of dairy cows. *MIR148A* is a domestication gene of dairy cows increasing milk yield. Estrogens, which are upregulated in pregnant dairy cows, stimulated the expression of miR-148a and miR-21, which attenuate the expression of DNA methyltransferase 1 (DNMT1). Various lipogenesis-inducing genes such as *sterol regulatory element binding factor 1* (SREBF1), *peroxisome proliferator*-*activated receptor*-*γ* (PPARG) and estrogen receptor-α (ERα). Procedures that increase dairy cow lactation performance are associated with an upregulation of lactation-promoting miRs, which may enter the human food chain in higher amounts

Epigenetic regulation of bovine MECs plays a crucial role in the biosynthesis of milk lipid and protein components. miR-148a targets key mediators involved in triacylglycerol and cholesterol homeostasis such as *ABCA1*, *LDLR* and *CPT1A* [403]. All members of the miR-148/152 family (miR-148a, miR-148b, and miR-152) share identical seed sequences [278, 404]. DNMT1 is a direct target of both miR-148a and miR-152 [68, 405]. The expression of miR-152 significantly increased during lactation in MECs of dairy cows producing high quality milk compared to lower miR-152 levels in cows producing low quality milk [406]. Forced expression of miR-152 in dairy cow MECs resulted in a marked reduction of DNMT1 at both the mRNA and protein levels [406]. In goat MECs, miR-148a induced milk triacylglycerol synthesis [407]. miR-148a expression can regulate *PPARA* and promoted triacylglycerol (TAG) synthesis while the knockdown of miR-148a impaired TAG synthesis in goat MEC. In addition, miR-148a cooperates with miR-17-5p to regulate fatty acid metabolism by repressing *PPARGC1A* and *PPARA* in goat MECs. Lactogenic hormones such as prolactin induce cellular and extracellular miR-148a expression in bovine MECs [408]. miR-148a belongs to the most abundantly expressed miRs of bovine milk since it accounts for more than 10% of the read counts in each stage of dairy cow lactation [409]. Directional selection of miR regulatory variants was important in the domestication and subsequent selection that gave rise to modern taurine cattle. The *MIR148A* gene has been identified as a candidate of domestication genes of modern cattle [410]. Co-expression network and pathway analyses identified bovine *MIR148A* as a major determinant enhancing milk yield [411]. Exaggerated miR-148a expression resulting in decreased DNMT1 expression is critical epigenetic change that induces lactation performance. Furthermore, persistent pregnancy of cows via increased E2 production may enhance E2-mediated miR-148a- and miR-21 expression in bovine MECs [333–335], thereby increasing the exosomal content of miR-148a and miR-21 (Fig. 15). In fact, increased miR-21 levels have been detected in skim milk of pregnant versus cyclic cows [354]. Thus, genetic selection of high performance dairy cows with enhanced miR-148a expression and pregnancy-dependent E2 production may be associated with an enrichment of miR-148a and miR-21 in milk exosomes enhancing the exposure of the human consumer of pasteurized milk to oncogenic miRs.

## Conclusion

Milk exosomes execute an evolutionary program under control of the lactation genome. They assist in the regulation of growth, tissue maturation, metabolic and immunological programming of the newborn infant. Obviously, this ancient exosome system has developed for maternal-neonatal communication and operates exclusively during the postnatal period of mammals except Neolithic humans who are persistently exposed to this system. The transfer of exosome-protected bovine milk miRs to human consumers has been considered to be one of the most important miR-based inter-species epigenetic communication related to the pathogenesis of human diseases [7–12, 412]. The restriction of milk exosomes to the postnatal period has been secured by physiological lactose intolerance that appears after weaning in all mammals except lactase (*LCT*)-mutated humans that could persistently abuse this postnatal epigenetic doping system. Unfortunately, the beneficial growth promoting and tissue-supporting effects of milk exosomes during early infancy may turn into obesigenic, diabetogenic, osteoclastogenic, cancerogenic and neurodegenerative signaling in adulthood. Dairy milk-derived exosomes should thus be regarded as potential pathogens that have spread unnoticeably into the human food chain. Technical progress with the introduction of pasteurization and refrigeration technology selected and preserved bovine milk exosomes, because the thermic conditions of pasteurization are not sufficient to eliminate bioactive milk exosomes [42–44]. Furthermore, pasteurization reduces exosome-degrading lactobacteria. Bacterial fermentation of milk attacks milk exosome proteins, reduces their size and miR content [48, 49].

As the milk exosome system is an archaic and highly conserved signaling system of all mammals [44], miRs exhibit a high degree of sequence homology [13, 44]. Remarkably, seed sequences of human and bovine miR-148a, miR-21, miR-29b, and miR-155 are identical (mirbase.org). Manca et al. [39] provided compelling evidence that bovine milk exosomes of pasteurized commercial milk reach the systemic circulation and distribute in tissues of different species. Efforts of dairy research to increase lactation performance and milk yield of dairy cows may have increased bovine miR-148a and miR-21 expression and their transfer to human milk consumer via milk exosomes [9].

Translational evidence supports the view that dairy milk exosomes are potential pathogens for human health (Table 1). This view is in accordance with epidemiological evidence showing adverse health effects for unfermented milk but no adverse or even beneficial health effects for fermented milk and milk products. Based on translational evidence, we conclude that milk exosomes should not reach the human food chain. Pasteurization of milk is an inappropriate method to prevent the spread of milk exosomes to the human milk consumer. In this regard, UHT is much more effective [43]. Other choices under recent investigation are microwave treatment or ultra-sonication of milk exosomes [39, 413].

**Table 1:** Translational evidence for dairy milk exosome-mediated pathologies of western diseases

Milk exosome component	Potential pathogenic involvement
miR-148a	Atherosclerosis, obesity, type 2 diabetes mellitus, hyperphagia, prostate cancer, breast cancer, hepatitis B-associated hepatocellular carcinoma, osteoporosis
miR-21	Adipogenesis, fetal macrosomia, prostate cancer, breast cancer, osteoporosis, hepatocellular carcinoma, diffuse large B-cell lymphoma
miR-29b	Type 2 diabetes mellitus
miR-155	Breast cancer, hepatitis C-associated hepatocellular carcinoma, diffuse large B-cell lymphoma, Parkinson’s disease, prostate cancer
TGF-β	Breast cancer, prostate cancer, osteoclastogenesis
Exosome lipids	Parkinson’s disease

Due to their low antigenicity, excellent bioavailability in many tissues and easy crossing of tissue boundaries such as the intestinal and blood–brain barrier, pharmacology became highly interested in bovine milk exosomes as therapeutic delivery systems of small interfering RNAs, drugs and phytochemicals [38, 414]. However, these new milk-exosome-based therapeutic options are a double-edged sword, because milk exosomes function as a Trojan horse not only transferring the new compound of interest but also the intrinsic exosome cargo such as oncogenic miRs and EMT-promoting TGF-β. Furthermore, it is of critical concern that exosomes may transfer viral RNA or DNA [415]. Novel replication-competent circular DNA molecules with potential proliferative activity have been detected in commercial milk of dairy cows [416–420], that via milk exosome transfer may reach distant tissue including the brain. Exosomes and their cargo are also involved in the spread of neurotoxic proteins such as α-syn, amyloid-β and prions [392, 393, 421–425]. Therefore, dairy milk-derived exosomes, although representing an easily accessible and abundant source of exosomes, are apparently not suitable for the treatment of human diseases. Milk exosomes meet the definition of bioactive food compounds and have an impact on human metabolism and gene regulation [426]. Before employing milk exosomes as drug delivery systems or supplements of infant formula, their unique intrinsic roles in the transmission of exosomal miRs and their potential ability to spread viral or neurotoxic pathogens require much more attention and most careful studies before introducing milk exosomes as carrier systems for the treatment of human diseases. Dairy milk exosomes should be regarded as potential new pathogens promoting western diseases.

## Abbreviations

ABCA1: ATP-binding cassette, subfamily A, member 1
ADRP: adipose differentiation-related protein
ALIX: apoptosis-linked gene 2-interacting protein X
α-syn: alpha-synuclein
AMACR: alpha-methylacyl-CoA racemase
BAT: brown adipose tissue
BC: breast cancer
BCAA: branched-chain amino acid
BCKD: branched-chain alpha-ketoacid dehydrogenase
BMD: bone mineral density
BMP: bone morphogenetic protein
BRCA1: breast cancer 1 gene
CaMKIIα: calcium/calmodulin-dependent protein kinase II alpha
CCK: cholecystokinin
CCK1R: CCK receptor 1 = CCK A receptor
CCK2R: CCK receptor 2 = CCK B receptor
CD63: melanoma-associated antigen MLA1
CD81: target of antiproliferative antibody (TAPA1)
CDKN1C: cyclin-dependent kinase inhibitor 1C (p57kip2)
CE: cholesteryl ester
C/EBP: CCAAT enhancer element binding protein
c-Fos: V-Fos FBJ murine osteosarcoma viral oncogene homolog
CRC: colorectal cancer
CSN1S1: casein alpha s1
DBT: dihydrolipoamide branched-chain acyltransferase
DLBCL: diffuse large B-cell lymphoma
DNMT: DNA methyltransferase
DOHaD: developmental origins of health and disease
EBV: Epstein Barr virus
ECM: extracellular matrix
ElF5: E74-like factor 5
EMT: epithelial–mesenchymal transition
ER: estrogen receptor
ERK: mitogen-activated protein kinase 4
EV: extracellular vesicle
FoxP3: forkhead box P3 (scurfin)
GLUT: glucose transporter
hASC: human adipose tissue-derived mesenchymal stem cell
HCC: hepatocellular carcinoma
HSP70: heat shock protein 70
HFD: high-fat diet
IL: interleukin
IFN: interferon
IRS-1: insulin receptor substrate 1
ITK: IL-2 inducible T-cell kinase
Kdm6b: lysine-specific demethylase 6b
LDL: low density-lipoprotein
LDLR: low density lipoptrotein receptor
MAFB: V-Maf musculoaponeurotic fibrosarcoma oncogene homolog B
MAPK: mitogen-activated protein kinase 1
MARCKS: myristolylated alanine-rich protein kinase C substrate
MEC: mammary epithelial cell
MFG: milk fat globule
miR: micro ribonucleic acid
MFGM: milk fat globule membrane
MSC: mesenchymal stem cell
mTORC1: mechanistic target of rapamycin complex 1
MV: microvesicles
MVB: multivesicular body
NPY: neuropeptide Y
NHL: non-Hodgkin lymphoma
PBM: peripheral blood monocytes
PBMC: peripheral blood mononuclear cell
PD: Parkinson’s disease
PCa: prostate cancer
PPAR: peroxisome proliferator-activated receptor
PPARGC1A: peroxisome proliferator-activated receptor-gamma, coactivator 1, alpha
RANKL: receptor activator of nuclear factor κB ligand
RASGRP1: ras guanyl nucleotide-releasing protein 1
rhPCR: RNase H2-dependent polymerase chain reaction
ROCK1: Rho-associated coiled coil-containing protein kinase 1
ROS: reactive oxygen species
SOCS: suppressor of cytokine signaling
SPARC: secreted protein acidic and rich in cysteine
SPRY2: sprouty 2
SREBF1: sterol regulatory element-binding transcription factor 1
STAT: signal transducer and activator of transcription
TAG: triacylglycerol
TC: total cholesterol
T2DM: type 2 diabetes mellitus
TGF-β: transforming growth factor-beta
TNF: tumor necrosis factor
TLR: toll-like receptor
Treg: regulatory T cell
TSDR: treg-specific demethylation region
TSG101: susceptibility gene-101
UHT: ultra-heat treatment
UTR: untranslated region
WAT: white adipose tissue
Wnt: wingless-type MMTV integration site family members
